# Space‐Charge‐Driven Nonlinear Charge Transport in Silicon Reconfigurable Nonlinear‐Processing Units

**DOI:** 10.1002/advs.78644

**Published:** 2026-11-09

**Authors:** Jonas Kareem, Lorenzo Cassola, Reinier J. C. Cool, Janiek I. van van Slooten, Ray J. E. Hueting, Peter A. Bobbert, Wilfred G. van der van der Wiel

**Affiliations:** ^1^ NanoElectronics Group MESA+ Institute, and Center for Brain‐Inspired Computing (BRAINS) University of Twente Enschede The Netherlands; ^2^ Institute of Physics University of Münster Münster Germany; ^3^ Power Electronics Group MESA+ Institute University of Twente Enschede The Netherlands; ^4^ Materials to Optoelectronic Devices and Eindhoven Institute for Renewable Energy Systems Eindhoven University of Technology Eindhoven The Netherlands

**Keywords:** nonlinear system, semiconductor device fabrication, semiconductor device modeling, silicon, space charge, transport phenomena, unconventional computing

## Abstract

Reconfigurable nonlinear‐processing units (RNPUs) are multi‐terminal electronic devices that act as computational primitives, exploiting intrinsic nonlinear charge transport combined with electrostatic tunability. Silicon‐based realizations provide a scalable and technologically relevant platform toward unconventional computing hardware, yet the physical origin of their room‐temperature nonlinearity has remained unresolved. Here, we demonstrate room‐temperature operation in both boron‐ and arsenic‐doped silicon RNPUs and show, using temperature‐ and length‐dependent measurements supported by TCAD simulations, that charge transport is governed by space‐charge effects. Interface trap states strongly suppress the equilibrium carrier density, while the functional nonlinearity arises from the competition between injected carriers and ionized dopants. The resulting transport evolves from Ohmic to strongly nonlinear and space‐charge‐limited current regimes, as evidenced by voltage and length scaling. The opposite‐polarity background doping is shown to control the onset and strength of the nonlinearity, producing behavior beyond the quadratic dependence of the classical Mott–Gurney law. Agreement between experiment and simulation supports that the spatial distribution of injected carriers and fixed charge governs the electric‐field profile and device response. These results establish a space‐charge‐based framework for RNPUs that does not require disorder or hopping transport, and provide design guidelines for scalable, CMOS‐compatible nonlinear computing hardware.

## Graphical abstract

Many approaches to AI hardware focus on accelerating linear operations, yet nonlinearity underpins computational expressivity. Reconfigurable nonlinear‐processing units (RNPUs) are promising computational primitives that provide nonlinear transformations directly in hardware. Using electrical measurements and semiconductor simulations, we show that their room‐temperature behavior is governed by space‐charge effects, establishing the operating principle of silicon RNPUs and design rules for CMOS‐compatible implementations.

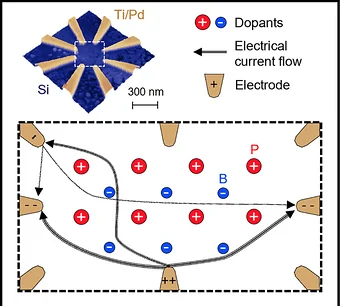

## 1 Introduction

In emerging computing hardware paradigms, including neuromorphic and broader unconventional electronics, nonlinear current‐voltage response and reconfigurability of conduction pathways are central to their functionality [1]. For example, resistive‐switching memristors rely on filamentary or trap‐mediated transport to achieve reconfigurable states [2, 3, 4, 5, 6], while reconfigurable transistors exploit tunable barrier, junction conduction, and charge trapping mechanisms, among others, for adaptive logic and memory functions [7, 8, 9, 10, 11, 12]. Unlike nonvolatile reconfigurable FETs and memory primitives that primarily switch the polarity, conductance, or logic state, silicon‐based reconfigurable nonlinear‐processing units (RNPUs) provide continuous multi‐terminal electrostatic control of a nonlinear transfer function within a CMOS‐compatible material platform (see Table S1 for a comparison with other reconfigurable devices). RNPUs similarly leverage intrinsic nonlinearity and tunability, enabling the solution of linearly inseparable classification problems, such as classification of the Modified National Institute of Standards and Technology (MNIST) digits [13], and ring classification and feature mapping tasks [14], as well as implementation in hardware neural‐network emulators [15]. More recently, they have served in temporal signal processing tasks like voice recognition [16] and as computing primitives in physical Kolmogorov‐Arnold Networks [17, 18]. By enabling programmable nonlinear transformations directly within a physical device, RNPUs provide compact analogue feature‐processing capabilities that may reduce the need for conventional multi‐stage computation and intermediate data movement in suitable architectures. Their realization in silicon and their room‐temperature operation furthermore provide a direct pathway toward CMOS‐compatible unconventional electronics.

Despite this progress, the underlying charge transport mechanism in silicon‐based RNPUs has remained insufficiently understood. Early RNPUs operated at cryogenic (liquid‐nitrogen) temperatures, and their behavior was interpreted in terms of hopping‐mediated transport in a disordered dopant network [13, 19, 20]. However, an unusually high dopant activation energy needed to be assumed to explain the temperature dependence of the charge transport [16, 21], suggesting that transport mechanisms other than the proposed 2D variable‐range hopping (VRH) via dopant states may be relevant. Recently demonstrated RNPUs [16] operating at room temperature further point toward transport mechanisms beyond hopping. While VRH can in principle occur via dopant states even in crystalline silicon [22, 23, 24], such mechanisms are typically most relevant at low temperatures, where carriers remain localized. At room temperature, thermally activated transport via extended states increasingly dominates over localized hopping pathways, making a hopping‐dominated interpretation less compelling for the present devices.

Here, we elucidate the origin of nonlinear charge transport in crystalline silicon RNPUs based on both boron‐ and arsenic‐doped devices through experimental current–voltage (*I*–*V*) characterization, including channel‐length and temperature dependence, complemented by commercial technology computer‐aided design (TCAD) simulations [25]. We show that space‐charge effects in the presence of interface states and, crucially, background doping are key to charge carrier transport at room temperature. Unless stated otherwise, the presented results focus on boron‐doped devices, while analogous behavior is observed in arsenic‐based RNPUs, confirming the generality of the proposed space‐charge‐driven transport framework across doping species. This insight enables the optimization of future device generations, including implementations aligned with standard CMOS processing. Importantly, we demonstrate that tunable nonlinearity and non‐monotonicity in *I*–*V* characteristics can be realized without invoking disorder‐mediated hopping, underscoring the potential for reproducible and scalable integration. The largely unexplored use of space‐charge effects in silicon for unconventional electronics points to a promising direction for such architectures.

## 2 Results

### 2.1 Room‐Temperature Nonlinearity and Functionality

We consider silicon‐based, 8‐electrode RNPUs, hereafter simply referred to as RNPUs, with either As or B ion implantation doping (Figure 1a and Figure S1) [13, 16]. We use standard (100) silicon wafers that are lightly doped (~10^15^ cm^−^^3^), with a doping type opposite to that of the implanted dopants (see Section 4.1). The ion implantation results in a doping gradient [13], with degenerate doping near the surface and a gradual decrease with increasing distance from the surface (Figure S2). Ohmic contacts are defined on the degenerately doped surface via electron‐beam lithography and metal evaporation. The devices are then wet‐etched in hydrofluoric acid (HF), which removes the native oxide; the silicon surface re‐oxidizes upon exposure to ambient conditions. The silicon is subsequently dry‐etched to reduce the doping (and hence the free carrier concentration) between the terminals, exploiting the decay of the doping concentration with depth (Figure 1b). We use reactive‐ion etching (RIE), which provides an anisotropic etch profile through the deposition of fluorocarbon species on the sidewalls (Figure S3). The metallic electrodes thus act as a hard mask, while the anisotropic etch preserves the Ohmic contacts. Thus, devices are fabricated within a larger implantation window (Figure S1), rather than using local contact implantation. More details on the fabrication of devices are provided in Section 4.1.

**FIGURE 1:**
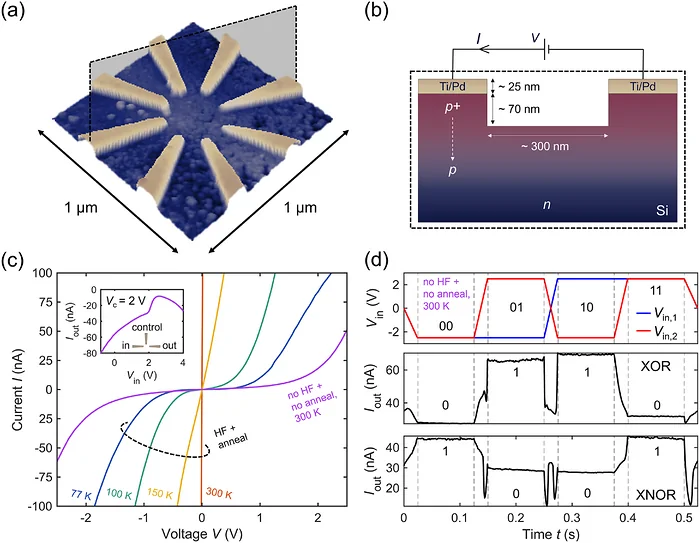
RNPU characteristics and logic functionality. (a) Atomic force microscope image of an RNPU. (b) Schematic cross‐section of a boron‐doped RNPU highlighting characteristic device dimensions. (c) Two‐terminal *I*–*V* characteristics of boron‐doped silicon reconfigurable nonlinear‐processing units (RNPUs), comparing untreated devices (no HF cleaning or annealing after dry etching, see text) with previously reported treated devices [13], at various temperatures. Untreated devices exhibit pronounced nonlinear behavior at room temperature, in contrast to the predominantly linear response of treated devices. Inset: An additional current‐carrying electrode (*V*_c_ = 2 V, *V*_out_= 0 V) enables non‐monotonic *I*–*V* characteristics. Additional (otherwise floating) electrodes further enhance tunability. (d) Untreated device demonstrating X(N)OR Boolean logic. Output current *I*_out_ measured in response to input voltages *V*_in,1_ and *V*_in,2_, which are applied onto electrodes adjacent to the electrode opposite to the output electrode.

Previously investigated cryogenic RNPUs operated at 77 K, while room‐temperature operation was achieved by applying a positive voltage to the lightly *n*‐doped substrate of boron‐implanted devices [13]. This was explained by the widening of the depletion region at the *p*‐*n* junction between the top layer and the substrate, accompanied by a suppression of band conduction. In the cryogenic RNPUs of Ref. [13], a post‐RIE HF etch was first applied to remove fluorocarbon residues resulting from RIE (Figure S4). However, this treatment leaves the silicon surface exposed to air, likely increasing the surface defect density [26], as reflected by the significant increase in resistance observed in the investigated devices following the standard HF cleaning step (Figure S5). A subsequent low‐temperature anneal (180–250°C) was therefore introduced to reduce the surface defect density [26]. Annealing increases the low‐bias conductance by more than 6000 fold relative to the post‐HF/pre‐anneal state, but the resulting conductance remains only approximately 2% of its pre‐RIE value. This leads to strongly Ohmic behavior at room temperature, requiring operation at lower temperatures to suppress the free carrier density.

By omitting the HF cleaning step, the low‐temperature anneal is no longer required, eliminating the need for cryogenic operation. This enables room‐temperature RNPUs with nonlinear *I*–*V* characteristics similar to those observed previously at lower temperatures (Figure 1c). By simply biasing an additional control electrode with voltage *V*_c_, the output current exhibits a complex dependence on the input voltage, including non‐monotonic *I*–*V* characteristics. This property is essential for RNPU functionality, as it introduces effective negative feedback (inhibitive response) [27, 28, 29, 30], giving rise to nonlinear transformations that can enable operations such as the XOR gate. A more detailed investigation of the multi‐terminal non‐monotonicity and device functionality will be presented elsewhere.

Accordingly, we demonstrate basic room‐temperature functionality by realizing linearly inseparable X(N)OR gates (Figure 1d), obtained by measuring the output current *I*_out_ for two input voltages *V*_in,1_ and *V*_in,2_. Compared to cryogenic RNPUs [13], we observe improved signal‐to‐noise ratio (SNR) and output‐state discrimination, enabled by the use of slightly higher control electrode voltages (up to 2.5 V) associated with the onset of strong nonlinearity. Other Boolean logic gates are also readily solvable (Figure S6). The demonstrated XOR/XNOR functionality provides one concrete example of how the electrically reconfigurable nonlinear response of the RNPU can be harnessed for computation. Importantly, the device reconfigurability can also support more complex analogue processing tasks [16, 17].

### 2.2 Role of Traps at Equilibrium

In both cryogenic [13, 21] and room‐temperature RNPUs (Figure 2a), we observe a distinctly high activation energy *E*_a_ for low‐bias transport between two opposite contacts, typically in the range of 0.3–0.4 eV for relevant etch depths (see Section 4.1). This suggests that their equilibrium transport mechanism shares a common origin. The resistance *R* in Figure 2a shows Arrhenius‐like behavior, characteristic of a thermally activated process:

(1) $$\begin{array}{c} R=R_{0}\exp\left(\frac{E_{a}}{k_{B}T}\right)\; \end{array},$$

with *R*_0_ a prefactor collecting all non‐activated contributions. We propose that the large *E*_a_ arises from a high density of surface states resulting in Fermi‐level pinning close to mid‐gap (Figure 2b). The surface damage induced by RIE is expected to significantly increase the density of dangling bonds at the silicon/native oxide interface, which act as traps. The predominant interface defects on (100) Si are *P*_b_ centers, namely *P*_b0_ and *P*_b1_ centers [31, 32], with *P*_b0_ typically dominating. Due to the amphoteric nature of these traps [33], introducing both donor and acceptor‐like states in the bandgap, both *n*‐type (arsenic‐implanted) and *p*‐type (boron‐implanted) devices exhibit high activation energies. The Fermi level pins near the charge‐neutrality level *E*_CNL_ [34], which depends sensitively on surface properties [35] and is likely governed by defect formation during and immediately after dry etching. These defects compensate the implanted dopants by trapping the free carriers they introduce, thereby significantly reducing the carrier concentration in the bulk of the device.

**FIGURE 2:**
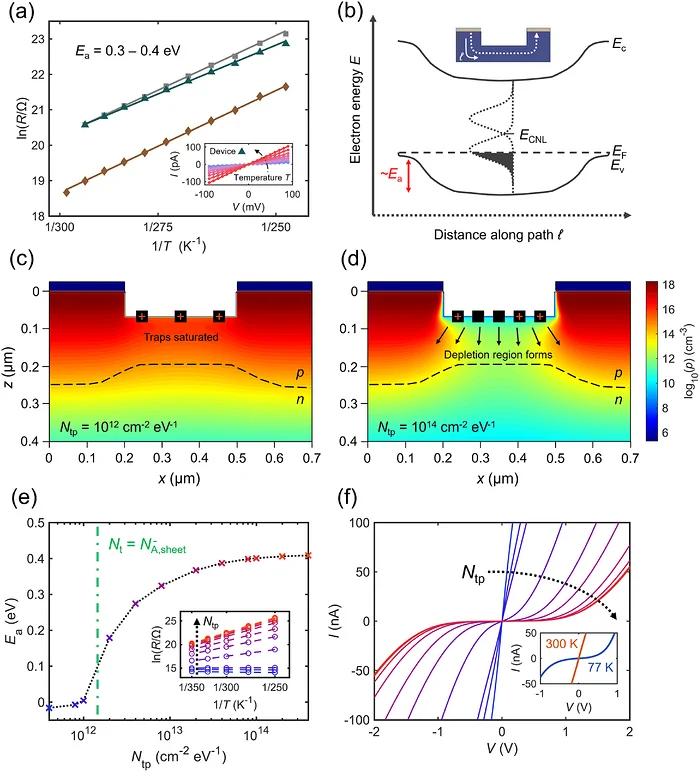
Trap‐induced reduction of the free carrier concentration. (a) Arrhenius plots of low‐bias resistance *R* (Ω) for boron‐implanted silicon RNPUs with different etch times: diamonds (1.5 min), squares (3 min), and triangles (4.5 min), with extracted activation energies *E*_a_. Inset: Low‐bias *I–V* characteristics of a single RNPU (triangle) vs. temperature (opposite contacts). (b) Equilibrium band diagram along current path l near the silicon surface (inset) in a boron‐implanted device. Surface trap states (see text) pin the Fermi level *E*_F_ (indicated by dashed line) near the charge‐neutrality level *E*_CNL_ between the conduction and valence band edges (*E*_c_ and *E*_v_), suppressing free carriers and forming a hole barrier with height *E*_a_. (c‐d) Technology computer‐aided design (TCAD) contour plots of free hole concentration for *N*_tp_ = 1.0 × 10^12^ cm^−2^ eV^−1^ and 1.0 × 10^14^ cm^−2^ eV^−1^, respectively. Traps at the Si/SiO_x_ interface are represented by black squares. Dashed line indicates the location of *p*‐*n* junction. (e) TCAD simulation of activation energy *E*_a_ as a function of peak interface trap density per unit energy *N*_tp_. The dash‐dotted line indicates the transition from a very low to a high *E*_a_. Inset: Arrhenius plots of low‐bias resistance *R* for increasing *N*_tp_. (f) Simulated *I–V* characteristics for increasing *N*_tp_. Beyond *N*_tp_ ~ 1 × 10^14^ cm^−2^ eV^−1^ the *I*–*V* characteristics do not change, in agreement with (e). Inset: *I–V* characteristics at 77 and 300 K for *N*_tp_ = 1.0 × 10^12^ cm^−2^ eV^−1^. Slight asymmetry in the *I*–*V* characteristics at 77 K is attributed to numerical error.

We confirm this scenario using Silvaco TCAD device simulations [25] (see Section 4.4), considering a 2D geometry that approximates a two‐terminal RNPU. Modelling the *P*_b_ centers as a pair of Gaussian trap distributions at the silicon/oxide interface (Figure S7) shows that the Fermi level is pinned near mid‐gap between the contacts (Figure 2b) and that the (equilibrium) carrier concentration is indeed significantly reduced in the etched region, extending from the surface into the bulk (Figure S8). The free hole concentration is approximately six orders of magnitude lower than expected from the implanted dopant concentration (Figure S2). At the silicon surface, both *n* and *p* approach the intrinsic carrier concentration *n*_i_ because the interface states capture carriers introduced by the dopants, causing the material to behave effectively as intrinsic silicon. Throughout the silicon depth, the mass‐action law

(2) $$\begin{array}{c} \mathrm{pn}=n_{i}^{2} \end{array}$$

holds, with the hole concentration peaking approximately 100 nm below the surface before decreasing again due to the implanted dopant profile. The exact carrier distribution depends on the capture cross‐sections of the defect centers, which in turn affects the probability of defect ionization *F*_t_. In steady‐state equilibrium, for the donor‐like states, this probability is given by

(3) $$\begin{array}{c} \;F_{t,D}=\frac{\sigma_{n,D}v_{n}n_{t,D}+\sigma_{p,D}v_{p}p}{\sigma_{n,D}v_{n}\left(n+n_{t,D}\right)+\sigma_{p,D}v_{p}\left(p+p_{t,D}\right)} \end{array},$$

with *σ*_n,D_ (*σ*_p,D_) the capture‐cross section of electrons (holes) for the donor‐like states, *v*_n_ (*v*_p_) the thermal velocity of electrons (holes), and *n*_t,D_ (*p*_t,D_) the concentration of electrons (holes) emitted from a donor‐like trap [25]. Consequently, the hole concentration reaches its minimum at the silicon surface, where carrier trapping is strongest.

We also performed low‐frequency noise (LFN) measurements and made use of Welch's method [36, 37] to investigate the trap dynamics (Figure S9). Assuming a noise power spectral density (PSD) with the behavior

(4) $$\begin{array}{c} S_{I}\left(f\right)=\frac{I^{\alpha }}{f^{\beta }}\;\;\left[10\right] \end{array},$$

where *f* is the frequency, and in the framework of the empirical Hooge model [38], we observe signatures of (de)trapping at low biases, including a distinct feature at 0.08 Hz, which we tentatively attribute to traps. At higher biases, the current dependence of the noise power deviates from the expected behavior for conventional 1/*f* noise with *α* = 2, instead exhibiting a much stronger dependence on current with *α* ≈ 5 (Figures S10 and S11). This nonlinear scaling may be indicative of a change in the dominant noise mechanism and, consequently, in accordance with the findings of Section 2.3, in the underlying conduction mechanism. The current noise at higher biases may also be related to mobility fluctuations potentially arising from impurity‐scattering effects [38].

In combination with the degenerately doped regions beneath the electrodes, theiw depletion of carriers creates a potential barrier between the contacts, analogous to a FET [39], with a height corresponding to the measured activation energy. In RNPUs, this barrier is not controlled by a capacitive gate, but by the electric field arising from the applied voltages and injected carriers. The distribution of interface states within the bandgap determines the position at which the Fermi level becomes pinned in the etched region and therefore directly controls the barrier height. Sufficiently high interface trap densities per unit energy, *N*_tp_, capture enough carriers in the etched region to establish a depletion region between the contacts, giving rise to a transport barrier whose height is determined by the pinned Fermi level. Conversely, when the total trap density is insufficient to compensate the charge associated with the implanted dopants, the Fermi level remains close to the valence band, preventing the formation of a significant depletion region and transport barrier (Figure 2c,d).

To quantify this effect, using TCAD simulations, we extracted the activation energy from the temperature dependence of the low‐bias resistance for different interface trap densities (Figure 2e). For low trap densities, the available interface states become saturated and are unable to significantly deplete the free‐carrier concentration. Transport is not thermally activated because the Fermi level remains close to the valence band, allowing carriers to diffuse and drift across the device with little impediment. As *N*_tp_ increases, the trapped charge progressively shifts the Fermi level away from the valence‐band edge in the etched region, increasing both the barrier height and the corresponding activation energy. At sufficiently high trap densities, the activation energy converges to ≈ 0.4 eV, indicating strong Fermi‐level pinning near the peak of the donor‐like trap distribution (*E*_v_ + 0.4 eV), beyond which further increases in trap density have little influence on transport (Figure 2f).

The transition from dopant‐dominated to trap‐dominated behavior occurs when the total interface trap density (for the donor‐like trap distribution in Figure S7)

(5) $$\begin{array}{c} \;N_{t}=\int_{E_{v}}^{E_{c}}g_{t}\left(E_{t}\right)\;dE_{t}\; \end{array}$$

becomes comparable to the sheet ionized acceptor density

(6) $$\begin{array}{c} N_{A,\mathrm{sheet}}^{\;-}=\underset{d_{\mathrm{etch}}}{\int^{\infty }}C\left(z\right)\;\mathrm{dz} \end{array},$$

a quantity that represents the effective surface charge density due to the implanted boron ions, where the concentration profile *C*(*z*) is taken from the etched surface. The etch depth, *d*_etch_ =  70 nm, is used in all TCAD simulations considered here. $N_{A,\mathrm{sheet}}^{\;-}$ is then 3.2 × 10^11^ cm^−2^, from which it is determined that *N*_tp_ =  1.5 × 10^12^ cm^−2^ eV^−1^ is the critical interface‐trap density per unit energy at the peak of the Gaussian trap distribution at which transport changes from dopant‐dominated Ohmic conduction to nonlinear, thermally activated behavior. Consistent with this picture, the room‐temperature *I*–*V* characteristics evolve from linear to strongly nonlinear as *N*_tp_ increases (Figure 2f). At lower trap densities (*N*_tp_ = 1.0 × 10^12^ cm^−2^ eV^−1^), the device may remain nearly Ohmic at room temperature but become strongly nonlinear at 77 K, as in the cryogenic RNPUs of Ref. [13], whereas for *N*_tp_ ~ 1 × 10^14^ cm^−2^ eV^−1^ pronounced nonlinearity is already present at room temperature.

These findings also explain the strong influence of surface treatment on device operation. HF etching followed by annealing reduces the interface‐trap density, resulting in Ohmic room‐temperature transport; nonlinear behavior can then only be recovered at lower temperatures, where thermal carrier generation is suppressed. In contrast, retaining the fluorocarbon layer preserves a high trap density, producing Fermi‐level pinning approximately 300–400 meV above the valence band and maintaining a sufficiently low free‐carrier concentration for nonlinear transport at room temperature. These results demonstrate that RNPU operation is governed by the balance between free carriers and trapped/depleted carriers, which together determine the emergence of the transport barrier and the resulting nonlinear behavior.

In contrast to boron RNPUs, arsenic RNPUs exhibit a stronger dependence on the etch depth (Figure S12). We attribute this difference to the nature of *P*_b1_ centers at the silicon/oxide interface, which lie slightly below mid‐gap, closer to the valence band edge [32]. The higher concentration of donor‐like trap states leads to a stronger Fermi level pinning for *p*‐type, hole‐transport devices, explaining the closely packed activation energies.

### 2.3 Non‐equilibrium Carrier Transport in RNPUs

In the following, we focus on hole‐transport devices, i.e., boron RNPUs. While the high activation energy establishes thermally activated, Ohmic transport at low bias, it does not account for the pronounced nonlinearity observed at higher bias. We therefore investigate the current–voltage characteristics over an extended bias range using dedicated test structures. Specifically, bar‐like transmission line model (TLM) structures [40] are fabricated on the implantation window, with the channel length *L* between the bars systematically varied (Figure 3a). The characteristic nonlinearity is observed for all channel lengths, with its onset shifting to higher voltages for longer channels, indicating a transport mechanism governed by the electric field. To identify the underlying transport mechanism, the *I*–*V* characteristics are analyzed on a log–log scale (Figure 3b), capturing all regimes from low to high bias. The degree of nonlinearity is quantified by the power‐law exponent *m* (Figure 3c), defined as

(7) $$\begin{array}{c} m=\frac{d\log\left(I\right)}{d\log\left(V\right)} \end{array}.$$

**FIGURE 3:**
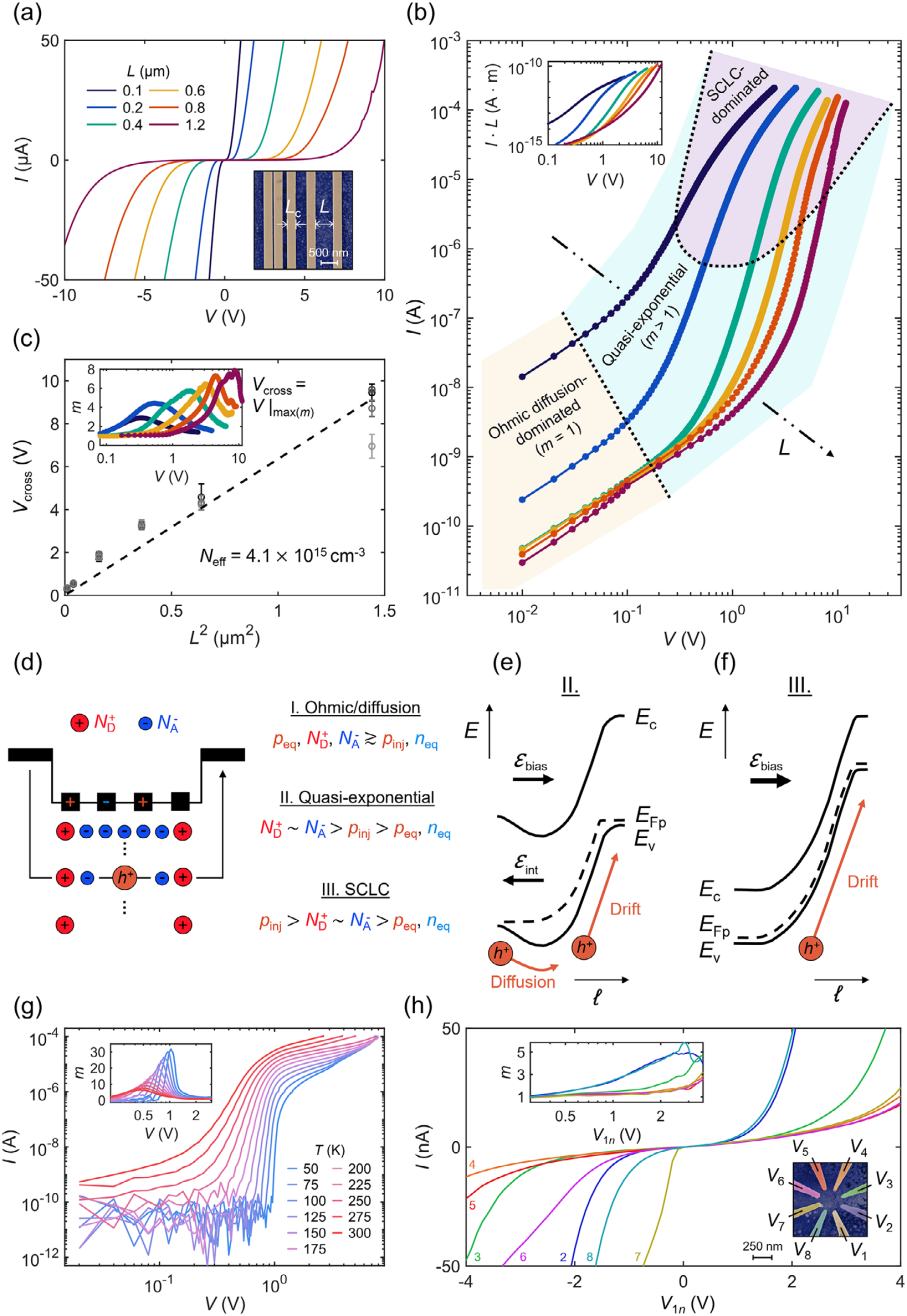
Experimental *I*–*V* characteristics of boron‐implanted (a)–(c),(g) transmission line model (TLM) structures and (h) RNPU. (a) *I*–*V* characteristics for increasing *L* with AFM image in the inset indicating channel (contact) length *L* (*L*_c_). (b) Log–log plot of *I*–*V* characteristics (etch depth = 70 nm, *L*_c_ = 200 nm) with three characteristic regimes indicated. Inset: *I*–*V* characteristics normalized to *L*. (c) Crossover voltages *V*_cross_ vs. *L* ^2^ for multiple structures (etch depth = 70 nm, *L*_c_ = 200 nm). Inset: Local power‐law exponent *m* vs. *V* for various *L*. *V*_cross_ corresponds to *V*|_max (*m*)_, i.e., the voltage at which *m* is maximal. (d) Schematic of relevant charge contributions governing hole transport, with hole current path highlighted. The charge‐balance conditions in the three transport regimes are indicated as inequalities. (e‐f) Schematic band diagrams along path l in the quasi‐exponential and SCLC‐dominated transport regimes, respectively. (g) Log–log plot of *I*–*V* characteristics at various *T* (*L* = 400 nm, *L*_c_ = 500 nm). Inset: *m* vs. *V* for various *T*. (h) Two‐terminal *I*–*V* characteristics of an RNPU. Current measured from biased electrode *V*_1_ to the grounded electrode *V_n_* (n=2,…,8), all other electrodes are kept floating. Top‐left inset: Local power‐law exponent *m* vs. *V* for different electrode pairs. Bottom‐right inset: False‐colored AFM image of an RNPU.

Following the Ohmic (*m* = 1) low‐bias regime, dominated by diffusion of dopant‐provided free carriers not trapped by surface states, transport becomes strongly superlinear (*m* > 1). As carriers are injected, the potential barrier between the contacts is progressively lowered. Under these non‐equilibrium conditions, transport in the active region can be described using Boltzmann (non‐degenerate) statistics, such that the valence band edge $E_{v}\left(l\right)$, and hence the barrier, shifts with the electrostatic potential according to

(8) $$\begin{array}{c} E_{v}\left(l\right)=E_{v0}\;-\left|e\right|\psi \left(l\right) \end{array},$$

where *E*_v0_ is its value at equilibrium electrostatic potential, |*e*| is the elementary charge, and ψ(l) is the position‐dependent electrostatic potential inside the device, determined by the applied voltages and the space‐charge distribution. The hole density is described by the quasi‐Fermi level *E*_Fp_, representing the effective chemical potential of holes under bias according to [39]:

(9) $$\begin{array}{c} p\left(l\right)=N_{v}\exp\left(-\frac{E_{\mathrm{Fp}}-E_{v}\left(l\right)}{k_{B}T}\right) \end{array},$$

with *N*_v_ the effective density of states in the valence band.

The *I*–*V* characteristics in the intermediate regime exhibit a strongly curved increase on a log–log scale (Figure 3b), which suggests an exponential dependence [41], as would arise from the Boltzmann statistics of Equation (9) under diffusion‐limited transport. Further analysis (Figure S13) shows that transport cannot be described purely by a diffusion current, though. This indicates that the implicit assumption of a linear relation between the electrostatic potential ψ(l) and the applied bias voltage *V* is not valid. The energy bands are nonetheless strongly modulated by *V*. TCAD simulations show that $E_{\mathrm{Fp}}-E_{v}\left(l\right)$ depends nonlinearly on *V* (Figure S14), demonstrating that the apparent exponential behavior does not arise from a pure diffusion current, but from a nonlinear electrostatic response. Diffusion, however, still contributes significantly, as discussed below.

We considered several charge transport mechanisms to explain the nonlinear non‐equilibrium behavior. In particular, for transport along interface states, both hopping (Figure S15) and Poole–Frenkel emission (Figure S16) were examined. However, the observed length scaling of the measured current, combined with TCAD results indicating that the dominant current path extends into the semiconductor volume rather than being confined to the interface, points to a bulk transport mechanism. Specifically, our analysis identifies space‐charge‐limited current (SCLC), which is known to dominate transport in *n* ^+^ *nn* ^+^ and *p* ^+^ *pp* ^+^ structures [42]. In planar geometries, the SCLC density *J*_SCLC_ follows a modified Mott–Gurney law with a weaker length dependence than the *L* ^−3^ dependence of the classical sandwich configuration [43, 44, 45, 46]:

(10) $$\begin{array}{c} J_{\mathrm{SCLC}}\propto \mu \varepsilon \frac{V^{2}}{L^{2}} \end{array},$$

where *μ* is the mobility and *ε* is the permittivity.

Moreover, at high electric fields, where velocity saturation sets in, the mobility in silicon becomes field‐dependent, often described by the Caughey–Thomas mobility model [47]:

(11) $$\begin{array}{c} \mu \left(E\right)=\mu_{0}{\left[\frac{1}{1+{\left(\frac{\mu_{0}E}{v_{\mathrm{sat}}}\right)}^{\alpha }}\right]}^{\frac{1}{\alpha }} \end{array},$$

with *μ*_0_ the low‐field mobility, E the magnitude of the lateral electric field (parallel to the current), *v*_sat_ the saturation velocity, and *α* a material‐dependent parameter on the order of 1. In the high‐field limit, SCLC modified by velocity saturation (VS) alters both the voltage and length dependence of the current density [48]:

(12) $$\begin{array}{c} J_{\mathrm{SCLC}-\mathrm{VS}}\propto v_{\mathrm{sat}}\varepsilon \frac{V}{L} \end{array}.$$

The inset of Figure 3b indicates an approximate *L* ^−1^ scaling of the current at high bias, which is consistent with SCLC in the velocity‐saturation regime. In addition, the slope plots in the inset of Figure 3c show that, in the high‐field limit, the voltage dependence approaches Ohmic behavior (*m* ≈ 1) for shorter channels. These findings are further supported by TCAD simulations: including field‐dependent mobility reproduces the observed channel‐length and voltage scaling, consistent with Equations (10) and (12) (Figures S17–S19). We thus find three distinct transport regimes: a low‐bias, diffusion‐dominated Ohmic regime (*m* = 1), a strongly nonlinear quasi‐exponential regime (*m* > 1), and an SCLC‐dominated regime, modified by velocity saturation at high fields (*m* ≈ 1). In the latter two regimes, transport is governed by space charge.

The presence of a space‐charge effect is supported by considering the charge contributions in the Poisson equation. The space‐charge effect occurs when the injected hole density exceeds both the equilibrium carrier density and the doping contributions [39]. Considering the charge density in a bulk region of the device, far from the contacts and assuming predominantly planar current flow, the relevant contributions are given by

(13) $$\begin{array}{c} \frac{\varepsilon }{\left|e\right|}\frac{dE}{\mathrm{dl}}=\left[\left(p\left(l\right)-p_{\mathrm{eq}}\right)-\left(n\left(l\right)-n_{\mathrm{eq}}\right)+N_{D}^{+}-N_{A}^{-}\right] \end{array},$$

where *p*_eq_ (*n*_eq_) is the equilibrium hole (electron) concentration, assumed constant in this volume, and $p\left(l\right)=p_{\mathrm{inj}}\left(l\right)+p_{\mathrm{eq}}$ and $n\left(l\right)=n_{\mathrm{inj}}\left(l\right)+n_{\mathrm{eq}}$ denote the total hole and electron densities, respectively, with *p*_inj_ and *n*_inj_ the concentration of the injected carriers. $N_{D}^{+}$ is the ionized donor concentration due to the phosphorus background doping, and $N_{A}^{-}$ is the ionized acceptor concentration due to the implanted boron dopants. Figure 3d schematically shows these contributions. In a hole‐transport device, both bulk and injected electron densities are negligible. Moreover, because interface states capture most free carriers near the central surface region, charge transport shifts into the bulk, such that $N_{A}^{-}∼N_{D}^{+}$ (Section 2.4). Because the degenerately doped contacts efficiently inject holes into the bulk, while interface traps strongly suppress the equilibrium carrier density in the etched region, the dominant competition is between $p_{\mathrm{inj}}\left(l\right)$, and the fixed ionized acceptors $N_{A}^{-}$ and donors $N_{D}^{+}$. The equilibrium hole density is relevant only in the low‐bias Ohmic regime, and is rapidly dominated by injected carriers at relatively higher bias. By suppressing the equilibrium carrier concentration, interface states enable space‐charge effects and the resulting SCLC behavior responsible for the observed nonlinear transport.

The injected holes are driven by the lateral electric field $E_{\mathrm{bias}}$ arising from the externally applied bias voltage *V*. Opposing this is an internal electric field $E_{\mathrm{int}}$, associated with the depletion‐induced transport barrier which counteracts the injection of holes. In this approximately exponential transport regime, the depletion regions formed near the surface and at the *p*‐*n* junction give rise to pronounced band bending (Figure 3e and Figure S14), steep carrier concentration gradients, and a strongly nonlinear lateral electric‐field profile. The diffusion current is therefore expected to contribute significantly to charge transport [41], further enhanced by the boron concentration gradient below the contacts. As the injected hole concentration increases and exceeds the net doping contribution, the injected carriers become the dominant contribution to the space charge. As a result, the externally applied field overwhelms the internal field, causing the transport barrier to collapse. Provided that the diffusion current is negligible compared to the drift current, transport then enters the space‐charge‐limited‐current regime (Figure 3f) [39]. Diffusion currents, which follow Boltzmann statistics in non‐degenerate semiconductors, are likewise important in neuromorphic silicon neuron circuits operating in the sub‐threshold regime [49, 50].

We define a crossover voltage *V*_cross_ separating the quasi‐exponential and SCLC regimes, corresponding to the condition $p>|N_{A}^{-}-N_{D}^{+}|$ across the device, analogous to the trap‐filled‐limit voltage in insulators with a bulk trap density [45]:

(14) $$\begin{array}{c} V_{\mathrm{cross}}=\frac{\sigma_{\mathrm{eff}}}{C_{0}}=\frac{eN_{\mathrm{eff}}L^{2}}{\varepsilon }\approx \frac{e|N_{A}^{-}-N_{D}^{+}|L^{2}}{\varepsilon } \end{array},$$

where *σ*_eff_ is the effective injected sheet charge required to satisfy $p>|N_{A}^{-}-N_{D}^{+}|$, *C*_0_ the areal capacitance between the electrodes, and *ε* = 11.8 *ε*_0_ [25] the permittivity of silicon. Here, *N*_eff_ denotes an effective carrier density, which approximates a net doping concentration. Additional carriers must be injected to overcome the hole density along the doping gradient beneath the contacts, as well as to compensate thermally generated carriers captured by interface traps in the etched region. As a result, the value of *N*_eff_ extracted from the crossover voltage is expected to overestimate $|N_{A}^{-}-N_{D}^{+}|$. *V*_cross_ thus corresponds to the bias voltage at which the total injected charge exceeds the fixed and trapped charge in the device, such that the electrostatics solely becomes governed by injected carriers, marking the onset of space‐charge‐limited transport. In order to extract *V*_cross_, we approximate that the peak voltage in the plot of *m*(*V*) (Figure 3c) is the voltage at which this onset occurs (Section 4.5). Fitting Equation (14) to the crossover voltages extracted from four test structures yields *N*_eff_ = 4.1 × 10^15^ cm^−3^, three orders of magnitude lower than expected for an etch depth of ~70 nm under the assumption $N_{\mathrm{eff}}=\left|N_{A}^{-}-N_{D}^{+}\right|$, based on the SIMS profile in Figure S2. The extracted value is instead much closer to the background phosphorus doping inferred from the substrate resistivity specified by the wafer supplier (Section 4.1), which lies in the range 5 × 10^14^ cm^−3^ and 5 × 10^15^ cm^−3^. In Section 2.4, we investigate the respective roles of the two doping contributions and show that the background doping is essential for the emergence of the quasi‐exponential regime.

With the dominant charge transport mechanism identified, we revisit the temperature dependence of the *I*–*V* characteristics for a 400 nm channel. As shown in Figure 3g, *V*_cross_ increases with decreasing temperature. This behavior can be understood from the reduction in *p*_eq_, which is governed by thermally activated generation of holes. As the temperature decreases, fewer holes are thermally excited, leading to a lower *p*_eq_. In addition, the high density of surface trap states within the band gap further suppresses hole activity, as trapped holes are less likely to be thermally re‐emitted at lower temperatures. As a result, the balance between $p\left(V\right)=p_{\mathrm{eq}}+p_{\mathrm{inj}}\left(V\right)$ and $|N_{A}^{-}-N_{D}^{+}|$ becomes temperature‐dependent: a lower *p*_eq_ requires a larger injected carrier density, which in turn necessitates a higher applied voltage. This leads to an upward shift of *V*_cross_, consistent with the observed temperature dependence. The pronounced temperature dependence of the current in the quasi‐exponential regime is consistent with established behavior [41, 46] and indicates that hole diffusion contributes significantly to transport in this regime. On the other hand, the weak temperature dependence in the high‐field regime is consistent with SCLC, where residual temperature dependence primarily arises from the carrier mobility [41].

Similar to hole‐transport devices implanted with boron, the analysis followed in Figure 3 can be extended to arsenic‐implanted devices and test structures. Figure S20a shows the *I–V* characteristics of an As TLM structure, from which we can perform a similar analysis, extracting the power‐law exponents and plotting *V*_cross_ vs. *L* ^2^ (Figure S20b). The power‐law exponent as a function of voltage reveals, similar to the boron‐implanted devices, a low‐bias Ohmic regime, a strongly nonlinear regime, and a transition toward a velocity‐saturation‐limited SCLC regime. Similar to the boron‐implanted devices, $N_{\mathrm{eff}}=3.5\times 10^{15}\;cm^{-3}∼\left|N_{A}^{-}-N_{D}^{+}\right|$, close to the resistivity of the boron‐doped wafers (5–10 Ω·cm), which corresponds to $N_{A}^{-}$ in the range 1 × 10^15^ to 3 × 10^15^ cm^−3^. The extracted *N*_eff_ lies closer to the median value of $N_{A}^{-}$ for these devices, in contrast to the difference observed between *N*_eff_ and $N_{D}^{+}$ in the boron‐implanted case. Besides variations in the implantation profiles (Figure S2), we attribute this primarily to differences in the contact geometry, represented by *L*_c_. Longer contacts promote an earlier onset of injected‐carrier‐dominated space charge in the semiconductor by enabling increased carrier injection (Figure S21). This reduces the crossover voltage and leads to an earlier onset of the SCLC regime (Figure 3c,g).

In RNPUs, due to the contacts becoming increasingly narrow as the active region of the device is reached (*L*_c_ ~ 20 nm), *V*_cross_ is higher compared to the test structures investigated here (Figure 3h). Within the measured voltage range, the crossover voltage is first reached by electrodes in close proximity, further emphasizing the channel‐length dependence of space‐charge phenomena. The voltage range relevant for RNPU operation lies entirely within the quasi‐exponential regime (< *V*_cross_), with *V*_cross_ (and the current) varying as a function of both voltage polarity and electrode separation. Under negative bias, holes are injected from the grounded electrode, resulting in a redistribution of positive space charge between the electrodes that differs from the case of positive bias. In addition, slight local variations in doping and trap density, as well as fabrication‐induced differences between contacts, can further influence the *I*–*V* characteristics. These observations underscore the additional complexity of charge transport in RNPUs arising from their electrode configuration and geometry. While the multi‐terminal geometry of RNPUs introduces additional electrostatic complexity, we argue that the underlying charge transport mechanism is the same.

### 2.4 TCAD Analysis of Background Doping in RNPUs

Silvaco TCAD device simulations [25] support the existence of three characteristic regimes, confirm the presence of space‐charge effects, and demonstrate the key role of background dopants in the nonlinear transport mechanism. Employing a two‐dimensional simulation geometry with varying channel length *L*, similar ∝*L* ^2^ scaling is obtained for different background doping concentrations (Figure 4a). The simulations also indicate that $N_{\mathrm{eff}}∼\left|N_{A}^{-}-N_{D}^{+}\right|$: for $N_{D}^{+}$ = 1.0 × 10^15^ cm^−3^, we obtain *N*_eff_ = 4.0 × 10^15^ cm^−3^, which is comparable to the experimentally extracted value. The background doping is essential to device functionality, as the depletion region associated with the *p*‐*n* junction shapes the internal electric‐field profile and thereby governs the onset of tunable nonlinearity with applied bias. Figure S22 shows that increasing the background doping shifts the onset of the SCLC regime to higher voltages, while nonlinearity is strongly suppressed when the background dopants are of the same type as the implanted dopants. In the latter case, the background doping increases the equilibrium hole concentration, and the *p*‐*n* junction with the substrate is effectively absent. Without this junction, the bulk depletion region does not form, leading to a higher hole concentration and weaker band bending associated with $E_{\mathrm{int}}$, which lowers the transport barrier for holes and suppresses the diffusion current contribution, effectively bypassing the quasi‐exponential regime. Strong nonlinearity therefore relies on depletion effects, which require the presence of dopants of opposite polarity to the injected carriers.

**FIGURE 4:**
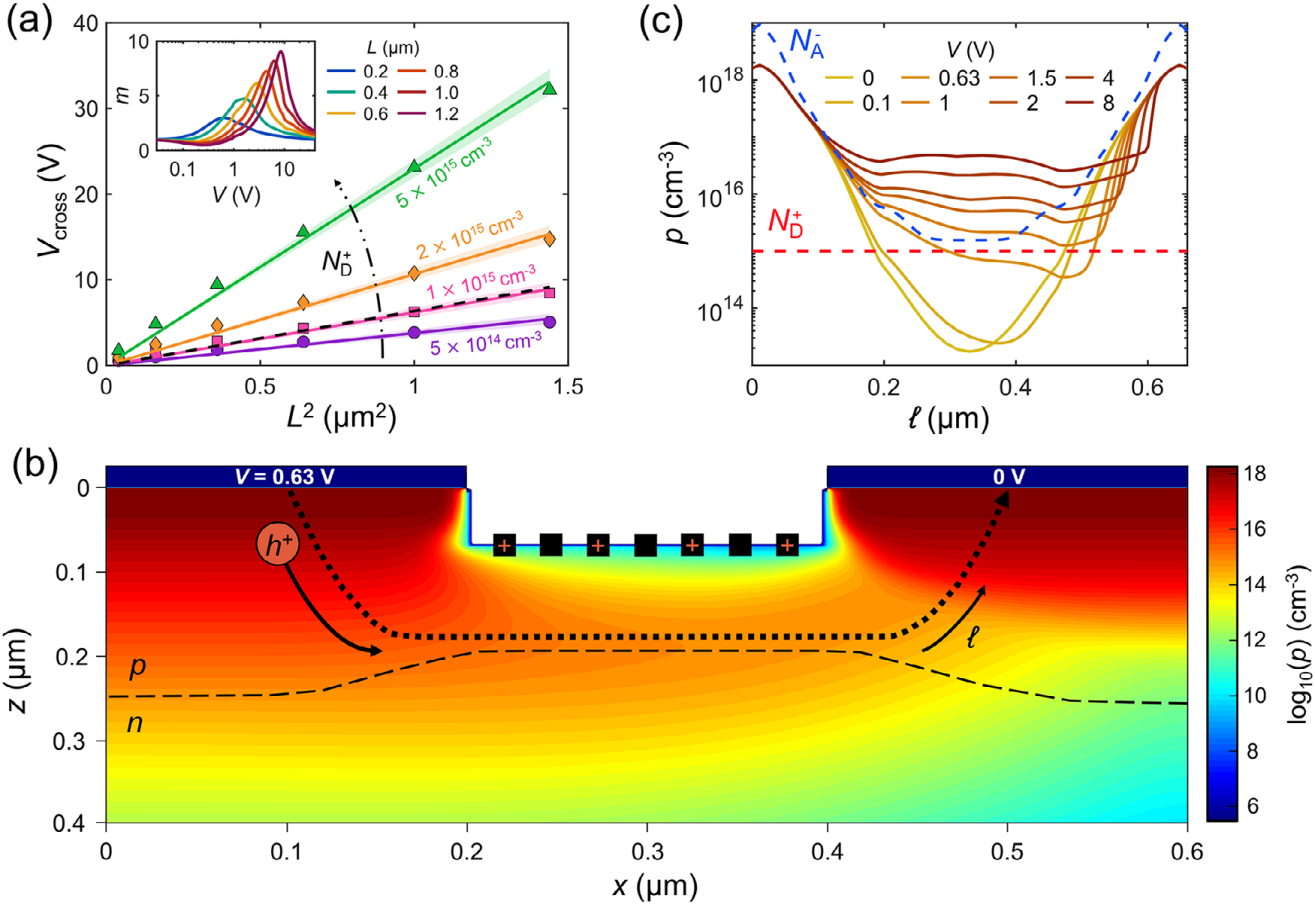
TCAD device simulations of a *p*‐type TLM structure, highlighting the role of background doping and traps. (a) *V*_cross_ vs. *L* ^2^ for different ionized donor doping concentrations $N_{D}^{+}$ (dot‐dashed arrow). Shaded regions indicate fit uncertainty; dashed line shows experimental fit from Figure 3c. Inset: Local power‐law exponent *m*(*V*) for varying channel lengths ($N_{D}^{+}$ = 1.0 × 10^15^ cm^−3^). (b) Contour plot of free hole concentration *p* with bias voltage *V* = 0.63 V ≈ *V*_cross_. Hole current flows along path l. Dashed line indicates the position of the *p*‐*n* junction. (c) Hole concentration along path l in (b) for increasing *V*. Red and blue dashed curves indicate $N_{D}^{+}$ and $N_{A}^{-}$, respectively.

Figure 4b shows a contour plot of the free hole concentration at a voltage approximately equal to *V*_cross_ = 0.63 V≈ 0.63 V, illustrating that the current flows in the bulk, several tens of nanometers beneath the silicon surface. The interface‐trap‐induced depletion region suppresses the free carrier concentration near the etched surface, forcing current flow deeper into the device, in the vicinity of the *p*‐*n* junction formed with the substrate. The Ohmic contacts and gradient in $N_{A}^{-}$ ensure efficient carrier injection and a low‐resistance path to the channel. As the injected hole density increases, the conductive region expands while the depletion region is pushed further into the bulk to maintain charge balance. Figure 4c depicts the hole concentration *p* along the cross‐sectional path l for different bias voltages, indicating that once *p* exceeds $|N_{A}^{-}-N_{D}^{+}|$ throughout the device (above ∼*V*_cross_), space charge becomes injection‐dominated, and transport enters the space‐charge‐limited regime. Below ∼*V*_cross_, steeper *p* gradients indicate a significant contribution of carrier diffusion to the current density, and hence to the device nonlinearity. Because the hole current penetrates slightly deeper into the semiconductor, the ionized acceptor concentration $N_{A}^{-}$ along this path becomes comparable to, and in some regions smaller than, *p* and $N_{D}^{+}$. The role of the ionized acceptors has thus far not been discussed in detail. If the boron SIMS profile is directly assumed to represent $N_{A}^{-}$, TCAD simulations do not reproduce the experimentally observed nonlinearity (Figure S23), instead yielding sub‐Ohmic *I*–*V* characteristics, likely due to mobility degradation from impurity scattering [25]. Reproducing the experimental behavior requires partial dopant deactivation within the channel. Reactive‐ion etching is known to electrically deactivate dopants through hydrogen diffusion and the formation of electrically inactive dopant complexes [51, 52, 53], particularly in hydrogen‐containing plasmas [51] such as the CHF_3_‐based plasma used here. Low‐temperature annealing near 450°C has been shown to restore dopant activity [52], suggesting that the annealing performed as part of the RNPU device treatment may simultaneously passivate *P*_b_ centers while reactivating electrically inactive dopants. More generally, independent of the underlying mechanism, the emergence of the quasi‐exponential regime requires a sufficiently low equilibrium hole concentration in the channel region to enable strong band bending. Both interface traps and electrically inactive (acceptor) dopants contribute to this reduction in carrier density, thereby enabling nonlinear space‐charge effects.

As discussed in Section 2.1, treated boron devices have been shown to be functional at room temperature by applying a positive potential to the *n*‐type substrate [13]. We can now explain this in the framework that we have presented. A positive substrate potential drives holes away from the bulk of the device toward the contacts, which are at a lower potential, widening the depletion region at the *p*‐*n* junction, thereby reducing *p*_eq_ in the bulk (Figure S24). This reduction enhances carrier concentration gradients and, consequently, the diffusion current, while shifting the onset of the SCLC regime to higher voltages. As a result, the slope *m* increases within the same voltage range, which is favorable for room‐temperature operation. These findings further indicate that the substrate potential can serve as an additional tuning parameter for untreated, room‐temperature RNPUs, particularly for controlling the quasi‐exponential regime prior to the onset of SCLC.

Notably, the voltage at which the injected hole concentration exceeds $|N_{A}^{-}-N_{D}^{+}|$ across the device corresponds closely to *V*_cross_ = *V*|_max (*m*)_ (Figure S14), but since it is based on a simplified 1D approximation, it may help explain deviations of the extracted *V*_cross_ from the ideal behavior given in Equation (14). Deviation between simulated and experimental crossover voltages may arise from a limitation of the two‐terminal TCAD model, which does not fully capture the influence of adjacent electrodes in the TLM geometry of Figure 3a. In the experimental structure, nearby contacts inject holes that extend into the channel region, increasing the local equilibrium hole density. As a result, a lower applied voltage (and hence fewer injected carriers) is required to reach the condition $p>|N_{A}^{-}-N_{D}^{+}|$. Both experimental and simulated *V*_cross_ vs. *L* ^2^ trends show that Equation (14) underestimates *V*_cross_, particularly for shorter channel lengths. This deviation likely results from the contact lengths becoming comparable to the channel length, thereby violating the assumptions of the ideal electrostatic model in Ref. [43]. Moreover, due to the bulk nature of the current flow, defining a particular voltage at which the injected carriers have overcome the equilibrium hole concentration and dopant concentrations is challenging. Figure 4b,c highlight this by showing that the space‐charge distribution, and thus the electric field profile, varies non‐uniformly across the device.

## 3 Conclusions

Using both boron and arsenic RNPU devices together with two‐terminal TLM structures that capture the relevant transport mechanism, we establish that space‐charge effects govern the charge transport underlying RNPU functionality in the devices studied here and in Ref. [13]. The modified fabrication route employed here, in which a previously used treatment step is omitted, enables robust room‐temperature operation. This shows that disorder‐mediated transport is not essential for their operation. Instead, the behavior is governed by two coupled charge balances. First, the interplay between RIE‐induced traps at the silicon/silicon oxide interface and implanted dopants sets the equilibrium carrier concentration. Sufficient suppression of this carrier density is required to enable tunable nonlinearity at room temperature, a condition that may be further enhanced by dopant deactivation. Second, once this condition is met, the competition between injected carriers and ionized dopants determines the dominant source of space charge. In general, a sufficiently low equilibrium carrier density in the active region allows strong band bending and the formation of an internal electric field (and associated transport barrier). In the present devices, the presence of oppositely doped regions provides a depletion region that further suppresses the hole concentration and establishes a spatially varying electric field, enabling the nonlinear and tunable response underlying device functionality.

While superquadratic SCLC behavior is typically associated with trap‐dense materials such as organics and perovskites [54, 55, 56, 57, 58], we show that RNPUs constitute an analogous system in crystalline silicon. Crucially, in contrast to these materials, the nonlinearity is governed primarily by dopants rather than traps. The observed *L* ^2^ scaling of the crossover voltage is analogous to the trap‐filled‐limit voltage, but arises from a fundamentally different physical origin. Because the mechanism is based on drift‐diffusion and electrostatics in an ordered system, device behavior is expected to be more predictable and reproducible, facilitating more consistent and transferable training across devices.

These insights provide a route toward planar silicon implementations with engineered doping profiles, reducing the reliance on etched structures and interface traps while strengthening compatibility with CMOS fabrication. Together with TCAD‐based modeling, this establishes a physics‐driven framework for optimizing RNPU devices and reducing device‐to‐device variation.

## 4 Methods

### 4.1 Device Fabrication

Fabrication of RNPUs follows the process flow described in Ref. [13]. After 26 µm × 60 µm implantation windows have been defined by wet etching into a thermally oxidized (100) silicon wafer (approximately 280 nm thick oxide), the exposed silicon is again oxidized to provide a stopping layer for ion implantation (25 and 35 nm thick for the As and B implants, respectively). In this way, the As and B peak concentrations are closer to the silicon surface. For the B implantation, the ion energy is 9 keV, and the dose is 3.5 × 10^14^ at/cm^2^, while for the As implantation the ion energy is 35 keV and the dose 1.0 × 10^14^ at/cm^2^. The B implantation is done on lightly phosphorus‐doped *n*‐type wafers with resistivities ranging between 1 and 10 Ω∙cm, whereas the As implantation is done on lightly B‐doped *p*‐type wafers with resistivities ranging between 5 and 10 Ω∙cm, as given by the supplier Okmetic. After 7 s rapid‐thermal annealing at 1,050°C and 1,100°C for the boron‐ and arsenic‐implanted wafers, respectively, the stopping layer is removed, markers are defined, and the wafers are diced into 1 × 1 cm^2^ samples. Both RNPUs and TLM structures are fabricated by means of electron‐beam lithography, electron‐beam evaporation, and a lift‐off process (Figure S1a,b). The metal structures consist of a ∼1.5 nm/25 nm Ti/Pd stack.

The implanted dopant profile, as determined by secondary‐ion mass spectroscopy (SIMS), is shown in Figure S2 for both B and As implants following rapid thermal annealing (RTA). It is possible that a fraction of dopants are inactive following the RTA step, though it is reported in Ref. [59] that for *T* ≥ 1,000°C all boron dopants are activated in ∼10 s for relevant doses. It is well‐known that dry etching can result in a severe reduction of dopant activity, and in some cases even complete deactivation, primarily attributed to hydrogen passivation of dopant impurities, which forms electrically inactive complexes [51, 52, 53]. Besides the surface states generated in the band gap by *P*_b_ centers, the dopant inactivity may thus also contribute to a significant reduction of the free carrier concentration. In our TCAD simulations, we assume that the fractions of active dopants are 20% below the contacts, and 5% in the channel region that is directly exposed to the plasma. We evaluate the sensitivity of the TCAD simulations to the fraction of inactive dopants, both below the contacts and in the channel regions (Figure S23).

The critical reactive‐ion etching (RIE) step is performed in a custom‐built parallel‐plate RIE reactor. We make use of a 5:1 CHF_3_/O_2_ mixture at a pressure of 25 mTorr, a substrate temperature fixed at 10°C, and an RF power equal to 25 W, driven at 13.56 MHz. This produces C_α_H_β_F_γ_ and F radicals to etch silicon (dioxide), with the C_α_H_β_F_γ_ radicals responsible for polymerizing the surface [60], in particular for mixtures containing fluorocarbon gases with γ/α < 3, which include CHF_3_ [61]. The polymer layer and sufficient oxygen ion bombardment lead to an anisotropic etching profile (Figure S3). After RIE, a thin polymer layer remains on the etched surface, providing partial protection to the silicon surface. Since the etch depth is determined via atomic‐force microscopy (AFM), it is thus underestimated unless the fluorocarbon layer is fully removed, e.g., by means of HF wet etching. This was part of the treatment followed in previous “treated” devices [13]. For the AFM measurements, a Bruker ScanAsyst‐Air silicon nitride tip is used, with a nominal tip radius of 2 nm on a silicon nitride cantilever.

Prior to RIE, the step height is determined, yielding the thickness of the metallic contacts (Figure S4a). The step height is extracted using the step height (positive) function, implemented in Gwyddion, a standard processing software for scanning probe microscopy data [62]. Following RIE (etch time = 3 min), the step height is measured again, from which an apparent etch depth of 40.8 ± 3.8 nm is extracted for one of the four TLM structures analyzed in Figure 3c. This apparent etch depth also yields optimal RNPU performance, providing strong nonlinearity while maintaining sufficient room‐temperature conductivity to enable Boolean logic gates with high fitness, i.e., clear separability between high and low states. The RNPUs in Figure 2a correspond to apparent etch depths of ≈25, ≈45, and ≈75 nm for etch times of 1.5, 3, and 4.5 min, respectively.

Following HF etching, we found that the real etch depth of the TLM structure is 70.3 ± 9.6 nm due to a 29.5 nm ± 10.8 nm thick fluorocarbon layer. This is also the etch depth used in our TCAD simulations, as it more accurately reflects the peak doping level in the silicon. Similar observations can be made on RNPUs (Figure S4b); however, due to the difference in geometry, the etch rate is reduced, and the deposited fluorocarbon layer is also slightly thinner. This is explained by the smaller feature sizes and correspondingly less silicon area in the active area of RNPUs, which affects how the radicals interact with the surface. Besides geometrical effects, the etch rates of boron‐ and arsenic‐implanted silicon also differ due to band bending effects at the surface [63]. The etch rate of arsenic‐implanted devices is higher than that of the boron‐implanted devices since the uncompensated As^+^ donors attract negatively charged F^−^ ions, enhancing the etch rate. In contrast, the etch rate is reduced by means of Coulombic repulsion for a silicon lattice containing B^−^ acceptor ions.

### 4.2 Electrical Characterization Methods

*I*–*V* characteristics at room temperature are measured in an MPI TS300‐SE Probe System with a Keithley 4200A‐SCS Parameter Analyzer. The substrate, which lies on the wafer chuck, is left floating for all measurements concerned here. One electrode remains grounded as the potential on the other electrode is varied from ‐*V* to *V*. For this reason, the log–log data that is plotted belongs to the positive range of the sweep since the current has adequately settled. Follow‐up measurements were performed to ensure consistency.

The temperature‐dependent measurements in Figure 2 were performed in a Suss MicroTec PM300 Probe System with a cooling unit, flushed with nitrogen under vacuum conditions. For the temperature‐dependent measurements that are plotted in Figures 1c, 3g, Figures S12 and S15, we use the PPMS Dynacool system, which makes use of evaporated liquid helium. This allows for characterization under cryogenic conditions, which is relevant for treated devices which operated at 77 K, and investigating hopping phenomena.

### 4.3 Evaluation of Device Functionality

We test our devices by wire bonding them onto custom PCBs, where typically seven electrodes are connected to DACs (cDAQ NI‐9264, 25 kS/s per channel) to serve as input and control electrodes. The remaining electrode is connected to a transimpedance amplifier (TIA) with a gain of 2 V/nA to convert the RNPU output current into a measurable voltage, which is subsequently digitized using an ADC (cDAQ NI‐9252, 50 kS/s per channel).

The solved Boolean logic gates in Figures 1d and Figure S6 are obtained by applying step voltages to the input electrodes, generating 10,000 random control voltage configurations, and measuring the current at the output electrode. The input voltages are applied such that they represent a 2‐bit logic circuit. They are applied to the contacts adjacent to the one opposite the output, and a set of control voltages capable of solving the desired task is found. Typically, a genetic algorithm is made use of in order to evolve the set of control voltages to obtain the highest possible fitness. The details of how this genetic algorithm is used for Boolean logic gate evolution are available in previous work [13, 64]. The output current ${\bar{I}}_{\mathrm{out}}$ for each input sequence is fitted with

(15) $$\begin{array}{c} {\bar{I}}_{\mathrm{out}}=m_{\mathrm{BL}}{\bar{X}}_{\mathrm{out}}+c_{\mathrm{BL}} \end{array},$$

where ${\bar{X}}_{\mathrm{out}}$ is the expected logic output (either 1 or 0), *m*_BL_ is a measure of the separation of the logic high and low levels, and *c*_BL_ is an offset term. The fitness of the Boolean logic gate is then evaluated by

(16) $$\begin{array}{c} \;F_{\mathrm{BL}}=\frac{m_{\mathrm{BL}}}{\sqrt{r_{\mathrm{ss}}}+k_{\mathrm{BL}}c_{\mathrm{BL}}} \end{array},$$

where *r*_ss_ is the fitting residual and *k*_BL_ an empirical constant. We choose *k*_BL_ to be 0.2 as in Ref. [13], and plot the Boolean logic gate waveforms with the highest fitness for each of the major logic gates in Figure 1d and Figure S6. In one run consisting of 10,000 configurations on the same device, it is possible to find configurations which use the same threshold (approximately 40 nA) for all gates, with the exception of the NAND gate.

### 4.4 Simulation Methods

Simulations are performed using Victory TCAD from Silvaco, a standard tool used in the semiconductor industry. The simulation comprises Victory Process (Version 8.42.5.R) and Victory Device (Version 1.24.0.R), which concern the fabrication‐ and device physics‐based aspects, respectively, of the simulation. For our purposes, we utilize Victory Process to generate a two‐dimensional, two‐terminal structure, as illustrated in Figure 4b. The simulations are thus a good approximation of the TLM test structure. This includes the assumption of a uniform background doping, as well as the generation of the mesh itself. The implantation profile of dopants opposite to the background doping is incorporated into the mesh using the concentration profile(s) shown in Figure S2. We then make use of Victory Mesh (Version 1.11.1.R) to reconstruct and transfer the structure to Victory Device. Alternatively, the profile can be implemented directly in Victory Device without Victory Process. The interface traps are assumed to be uniform along the entire silicon/silicon oxide interface. We assume *P*_b0_ centers are the dominant contribution to the surface states in the bandgap, represented by two Gaussian distributions, one acceptor‐like (in the vicinity of the conduction band) and one donor‐like (in the vicinity of the valence band). The density of states *g*_t_ of such a Gaussian trap distribution is given by

(17) $$\begin{array}{c} g_{t}=N_{\mathrm{tp}}\exp\left[-{\left(\frac{E_{t}-E_{\mathrm{tp}}}{W_{t}}\right)}^{2}\right] \end{array},$$

where *N*_tp_ is the trap density per unit energy at the peak of the Gaussian, *E*_tp_ is the energy level at which the Gaussian peaks (relative to the respective band edge), *E*_t_ is the energy level of the trap state, and *W*_t_ is the characteristic decay energy of the Gaussian [25]. We assume two identical distributions, with *N*_tp_ = 2 × 10^14^ cm^−2^ eV^−1^, *E*_tp_ = 400 meV, and *W*_t_ =  120 meV (Figure S7). The distributions were placed such that they were near mid‐gap in accordance with Refs. [31, 32], with the actual density being chosen such that sufficient traps exist to pin the Fermi level (Figure 2e). We expect a density of interface states several orders of magnitude above typical interface state densities of high‐quality interfaces (*D*_it_ ~ 10^10^ − 10^11^ cm^−2^ eV^−1^) [65] due to the unannealed, RIE‐damaged surface.

The simulation is based on a few fundamental physical equations; the equations are given as described in Ref. [25]. Computations are performed using Maxwell‐Boltzmann statistics, so that the probability of a state with energy *E* being occupied is given by

(18) $$\begin{array}{c} F\left(E\right)=\frac{1}{1+\exp\left(\frac{E-E_{F}}{k_{B}T}\right)}\;\approx \exp\left(-\frac{E-E_{F}}{k_{B}T}\right) \end{array}.$$

We found that when instead using Fermi‐Dirac statistics, the computed *I–V* characteristics were identical, supporting the assumption that *E* − *E*_F_ ≫ *k*_B_ *T* and thus justifying the use of Boltzmann statistics, which reduces computation time. The electrostatics in the device is described by Poisson's equation, with the device simulator considering five overall contributions [25]:

(19) $$\begin{array}{c} \nabla \cdot \left(\varepsilon E\right)=\rho =\left[\left|e\right|\left(p-n+N_{D}^{+}-N_{A}^{\;-}\right)+Q_{T}\right] \end{array},$$

whereE is the electric field, *ρ* is the bulk charge density, and *Q*_T_ is the charge density contribution due to traps. The contribution due to traps is computed based on the integral of the density of states and the trap occupancy:

(20) $$\begin{array}{c} \;Q_{T}=\left|e\right|\underset{E_{v}}{\int^{E_{c}}}\left[g_{t,D}\left(E_{t}\right)\cdot F_{t,D}\;-\;g_{t,A}\left(E_{t}\right)\cdot F_{t,A}\right]dE_{t} \end{array},$$

where we consider both donor‐like and acceptor‐like distributions, with *F*_t,D_ (*F*_t,A_) the probability of defect ionization for the donor‐like (acceptor‐like) states, as described by Equation (3).

The carrier transport equations

(21) $$\begin{array}{c} J_{n}=|e|n\mu_{n}E_{n}+\left|e\right|D_{n}\nabla n \end{array}$$

and

(22) $$\begin{array}{c} J_{p}=|e|p\mu_{p}E_{p}\;-\left|e\right|D_{p}\nabla p \end{array}$$

are based on the drift‐diffusion model [25], with *D*_n_ (*D*_p_) the diffusion coefficients of electrons (holes). When space‐charge‐limited currents dominate charge transport, the second term, i.e., the diffusion current, is negligible compared to the drift current contribution. The current density, as well as the assumed Shockley‐Read‐Hall‐like recombination, are central to the carrier continuity equations [25]:

(23) $$\begin{array}{c} \frac{\partial n}{\partial t}=\frac{1}{\left|e\right|}\nabla \cdot J_{n}+\left(G_{n}-R_{n}\right) \end{array}$$

and

(24) $$\begin{array}{c} \frac{\partial p}{\partial t}=-\frac{1}{\left|e\right|}\nabla \cdot J_{p}+\left(G_{p}-R_{p}\right) \end{array},$$

with *G*_n_ (*G*_p_) and *R*_n_ (*R*_p_) the generation and recombination rates for electrons (holes). These equations comprise the system of coupled, nonlinear partial differential equations which are discretized over the mesh using the finite‐element method. The equations are then solved iteratively using the Newton method. The boundaries of the simulation use Neumann boundary conditions, i.e., the current is set to zero [25]. In our simulations, rather than assuming all dopants to be ionized, which is a good assumption at room temperature, the incompletely ionized dopant concentrations are calculated and used when determining the charge densities.

Finally, mobility models are used in order to more accurately capture the measured *I–V* characteristics, in particular with increasing bias, which leads to a field‐dependent mobility. We use the Lombardi CVT model [66] for the transverse‐field dependent component of the mobility, the Klaassen model for the bulk mobility [67, 68], which is in particular relevant for impurity scattering effects in a large concentration range [25], and the Caughey–Thomas saturation‐velocity dependent mobility model for the parallel‐field component, as given by Equation (11).

### 4.5 Crossover Voltage Extraction

As discussed in Section 2.3, the peak voltage in the *m*(*V*) characteristics is used to extract the crossover voltage *V*_cross_. The quantity *m*(*V*) is obtained by locally estimating the power‐law exponent via a sliding‐window fit. Specifically, least‐squares linear regression is performed in a window of size *M* in log *I*−log *V* space, which is sequentially shifted across the data. This yields a set of local slopes *m*, each associated with an averaged voltage

(25) $${\bar{V}}_{i}=\begin{array}{c} \frac{1}{M}\sum_{k=0}^{M-1}V_{i+k} \end{array},$$

where *i* denotes the index of the measured *I* −*V* data. For the extraction of *V*_cross_ in Figures 3c, 4a, and Figure S20b, we use *M* = 2 to maximize sensitivity to the local power‐law behavior. An exception is one structure in Figure 3c, for which *M* = 5 is used to mitigate noise. For all other *m*(*V*) curves shown for visualization purposes, *M* = 2 is employed, except in Figure 3g,h, where *M* = 20 and *M* = 5 are used, respectively, to further suppress noise.

### 4.6 Statement on the Use of AIGC Tools

We have solely used ChatGPT for language and code improvement.

## Author Contributions

**Jonas Kareem**: conceptualization, investigation, validation, methodology, writing – review and editing, writing – original draft, visualization, data curation, formal analysis, software. **Lorenzo Cassola**: conceptualization, investigation, writing – review and editing, formal analysis. **Reinier J. C. Cool**: conceptualization, investigation, writing – review and editing, formal analysis. **Janiek I. van Slooten**: conceptualization, investigation, writing – review and editing, formal analysis. **Ray J. E. Hueting**: resources, writing – review and editing, validation, methodology. **Peter A. Bobbert**: writing – original draft, writing – review and editing, formal analysis, methodology, conceptualization. **Wilfred G. van der Wiel**: writing – review and editing, writing – original draft, funding acquisition, resources, supervision, project administration, conceptualization.

## Funding

This publication is part of the project NL‐ECO: Netherlands Initiative for Energy‐Efficient Computing (with project number NWA. 1389.20.140) of the NWA research programme Research Along Routes by Consortia, which is financed by the Dutch Research Council (NWO). We acknowledge financial support from the HYBRAIN project funded by the European Union's Horizon Europe research and innovation programme under grant agreement no. 101046878. This work was further funded by the Deutsche Forschungsgemeinschaft (DFG, German Research Foundation) grant no. SFB 1459/2 2025–433682494.

## Conflicts of Interest

The authors declare no conflicts of interest.

## Supporting information




**Supporting File**: advs78644‐sup‐0001‐SuppMat1.docx.

## Data Availability

All data and code underlying this study are openly available [69].
