# Supplementary material for: Space‐Charge‐Driven Nonlinear Charge Transport in Silicon Reconfigurable Nonlinear‐Processing Units
Source: Adv Sci (Weinh). 2026 Nov 9:e78644. Online ahead of print. doi: 10.1002/advs.78644 (PMC13749006; doi:10.1002/advs.78644)
Supplement: Supplementary file 1 [file ADVS-9999-e78644-s001.docx]

**Supporting Information**

for

**Space-Charge-Driven Nonlinear Charge Transport in**

**Silicon Reconfigurable Nonlinear-Processing Units**

J. Kareem^1*^, L. Cassola^1^, R.J.C. Cool^1,2^, J.I. van Slooten^1^, R.J.E. Hueting^3^, P.A. Bobbert^1,4^, W.G. van der Wiel^1,2†^

^1^*NanoElectronics Group, MESA+ Institute, and Center for Brain-Inspired Computing (BRAINS), University of Twente, P.O. Box 217, 7500 AE Enschede, The Netherlands*

^2^*Institute of Physics, University of Münster, 48149 Münster, Germany*

^3^*Power Electronics Group, MESA+ Institute, University of Twente, P.O. Box 217, 7500 AE Enschede, The Netherlands*

^4^*Materials to Optoelectronic Devices and Eindhoven Institute for Renewable Energy Systems, Eindhoven University of Technology, P.O. Box 513, 5600 MB Eindhoven, The Netherlands*

^*^Contact author: jonas.kareem@utwente.nl

^†^Contact author: w.g.vanderwiel@utwente.nl

**Table S1.** Comparison of emerging reconfigurable devices in terms of operating mechanism, operating voltage, device footprint and tunability

| **Platform** | **Reconfiguration mechanism** | **Operating voltages** | **Characteristic active-area dimensions** | **Tunability** |
| --- | --- | --- | --- | --- |
| **This work, Si RNPU** | Multi-terminal electrostatic modulation of the space-charge profile | Typically max. ±2.5 V | Electrode separations of 50–300 nm (active area); effective transport depth of a few hundred nm | Continuous tuning of the nonlinear transfer function |
| **Lee et al., Nat. Commun. (2023)** [1] | Floating-gate charge modulation of carrier injection in an organic heterojunction transistor | Programmingvoltages: -15 V / +18 V. Source-drain voltage up to 6 V | Lateral (channel) dimensions are 400 x 800 µm². Stack thickness ~ 250 nm | Electrically switches between negative trans-conductance (NTC) and monotonic transfer characteristics, enabling binary/ternary logic reconfiguration |
| **Berdan et al., Nat. Electron. (2020)** [2] | Nonlinear tunnelling in ferroelectric tunnel junction (FTJ) memristors; polarization-controlled resistive switching | Read voltages up to 3 V, write voltage: 5 V | 5-nm-thick device layer thickness; lateral dimensions between 150–500 nm | Nonvolatile, multilevel conductance states; nonlinear response supports low-current, linear analogue VMM |
| **Günkel et al., ACS Appl. Electron. Mater. (2026)** [3] | Trap-controlled space-charge-limited conduction and field-induced metal-insulator transition in an oxygen-deficient YBCO interfacial layer | Read and write voltages investigated from ±1–3 V | 80 x 80 µm² active YBCO island; 30–100 nm film thicknesses | Nonvolatile bipolar resistive switching |
| **Zhu et al., Nat. Commun. 2026** [4] | Charge trapping in an Al₂O₃/HfO₂/Al₂O₃ stack in a MoTe₂-based vdW RFET | Programming voltages up to ±15 V; *V*_DD_ = 1–3 V | Flake lateral dimensions of ~tens of µm; multilayer stack thickness of 50–100 nm | Nonvolatile polarity switching, symmetric n-/p-type operation, and 6-bit nonvolatile conductance states |
| **Peng et al., Nat. Electron. 2023** [5] | Programmable graded doping in MoTe₂ via molecular adsorption/  desorption | Source–drain and bottom-gate voltages > ±10 V (maximum 28 V) | Channel length of 1.4 µm, width of a few µm; MoTe₂ thickness of ~5 nm | Polarity-switchable p–n junctions, memory, Boolean logic, and synaptic functions |
| **Lee et al., Sci. Adv. 2023** [6] | Photo-controlled charge trapping in a MoTe₂-based Si–vdW heterostructure | *V*_DD_ down to 0.5 V; bottom-gate voltage up to ± 80 V | Flake lateral dimensions of ~tens of µm; transport depth of ~50 nm | Nonvolatile polarity switching, symmetric n-/p-type operation, and logic reconfiguration |

**Figure S1.** Schematic overview of (a) RNPU and (b) TLM designs. Devices are defined on 26 μm $\times$ 60 μm implantation window (blue). The 150 μm $\times$ 150 μm bonding pads are metalized on 280 nm thick thermally grown silicon oxide (green) covering most of the silicon substrate.

**Figure S2.** Boron (red) and arsenic (blue) implantation concentration $C$ profiles as a function of depth $z$, determined via secondary-ion mass spectroscopy (SIMS). The boron dopant profiles used in TCAD simulations for below the contacts (dashed line) and for the channel (dotted line) are also shown, assuming that an effective constant fraction of dopants is inactive. SIMS measurements and analysis performed by tascon GmbH.

**Figure S3.** Cross-sectional schematic of the reactive-ion etching (RIE) process. C_α_H_β_F_γ_ radicals form a fluorocarbon polymer layer, while oxygen ion bombardment removes this layer from the horizontal (100) surface. Polymer passivation is therefore retained mainly on the sidewalls, protecting them from chemical etching and enabling an anisotropic etch profile.

**Figure S4.** (a) Atomic-force-microscope (AFM) cross-section of a TLM structure, showing the measured step heights before RIE, after RIE, and after wet etching in hydrofluoric acid (HF). Based on the difference in step heights before and after HF etching, a 29.5 nm ± 10.8 nm thick fluorocarbon layer is likely deposited on the (100) silicon surface. (b) Height profile across RNPU contacts, where the average step height taken over eight contacts is plotted. The smaller step height difference relative to the TLM structure indicates that the reactive-ion etching is geometry-dependent, with an estimated fluorocarbon layer thickness of 13.5 ± 5.0 nm. The deviations from the baselines for the smaller gap sizes in the TLM structure is attributed to edge overshoot [7].

**Influence of HF wet etch on electrical properties**

The influence of the HF wet etch on the electrical properties of the device is an order-of-magnitude conductance reduction at moderate bias voltages and a strong suppression of the nonlinearity for the measured voltage range (Figure S5). The electrically active carrier concentration appears to be substantially reduced, possibly due to exposure of the silicon surface, which increases the density of dangling-bond-related surface states and enhances carrier trapping. This shifts the onset of strong nonlinearity to higher voltages, where device breakdown may occur before any functionality is attained. For an etch depth of 70 nm, and ~70 nm electrode spacing (adjacent electrodes), the breakdown voltage is estimated to be ~5.3 V, using the critical breakdown field of silicon of $2.5\times{10}^{5} V/cm$ [8]. Given the gradient in the doping and the influence of interface states on the free carrier concentration, space-charge effects – and the resulting nonuniform electric field – cannot be neglected, and may significantly alter the critical breakdown field of the device. Nevertheless, the device resistance exceeding giga-ohms, the resulting low signal-to-noise ratio, and the low degree of nonlinearity for typical voltages have consequences for both readout and device functionality. This necessitates annealing in order to lower the resistance, however, as reported in Ref. [9] and in Figure 1c, this in turn requires lowering the temperature to suppress the free carrier concentration such that the strong nonlinearity induced by space-charge effects can re-emerge. These experimental observations are supported by TCAD simulations, which show that for a given trap density the *I*–*V* characteristics are linear at room temperature and become nonlinear at 77 K (Figure 2f). The conductance is, however, only partially restored relative to the pre-RIE state.

**Figure S5.** Comparison of *I*–*V* characteristics of a boron-implanted RNPU after RIE and after the HF wet etch (right axis), overlaid against the *I*–*V* characteristics of two other devices measured before RIE and following annealing (left axis). The bias voltage is swept on one input electrode, the neighboring output electrode is grounded, and the remaining electrodes are left floating. The low-bias conductance (fit range: ±200 mV) extracted for each device state is included in the legend.

**Figure S6.** Remaining Boolean logic gates solved on same untreated device in Figure 1d. Using Equation (16), the calculated fitness values of the (a) AND, (b) OR, (c) NAND and (d) NOR gates are 9.3, 8.5, 0.4 and 6.2, respectively.

**Figure S7.** Trap distributions used in TCAD [10] at the silicon/silicon oxide interface to emulate $P_{b}$ centers, approximated here by $P_{b0}$-like states [11, 12].

**Figure S8.** Simulated electron and hole concentrations versus depth $z$ in the center of *p*-type TLM structure ($L=300 nm, L_{c}=200 \mathrm{nm}$) at equilibrium. The depth begins at the etched silicon surface with the etch depth equal to 70 nm.

**Low-frequency noise measurements**

We use the measurement setup described in Section 4.3 to characterize the low-frequency noise between two electrodes of the RNPU. For each measurement, a fixed dc bias voltage is applied and the resulting output current is recorded as a time-domain signal. After a 60-second settling time, the current is sampled over $t=1000 s$ at a rate of 10 kS/s (10^7^ samples) and the resulting time trace is used to calculate the current-noise power spectral density (PSD). The PSD is obtained using Welch’s method [13], in which the time-domain signal is divided into overlapping 100 s long segments (50% overlap), each segment is multiplied by a Hann window, and the corresponding periodograms are calculated and averaged. This procedure reduces the variance of the spectral estimate while retaining the frequency-dependent noise characteristics of the device. The resulting noise spectra for increasing bias voltages are shown in Figure S9a.

The noise spectra are subsequently normalized to the current to examine how the relative noise amplitude evolves with current (Figure S9b). A distinct feature at approximately 0.08 Hz emerges only below a bias voltage of ~1.8 V, corresponding to a current of ~5 nA. At lower currents, the amplitude of this feature, together with the low-frequency 1/*f* noise, exhibits an $I^{2}$ dependence on the current.

Plotting $S_{I}$ as a function of current at fixed frequencies (Figure S10) reveals a distinct change in the current dependence at approximately 5 nA. Below this current, the noise PSD follows a quadratic current dependence, whereas at higher currents the PSD shows a substantially stronger dependence on current. This crossover coincides with the disappearance of the 0.08 Hz feature from the measured spectra. To quantify the frequency and current dependence of the noise, we assume that the PSD can be described by Equation (4). The resulting estimates of *α* and *β* over different current and frequency ranges are shown in Figure S11. At low frequencies and low bias voltages, the extracted exponents are consistent with ideal 1/*f* noise, for which *α* = 2 and *β* = 1. However, the measured noise in this regime may include contributions from the measurement electronics, including the ADC, DAC and TIA. In contrast, *α* differs substantially between the low- and high-current regimes, with values of approximately 2 and 5, respectively. The gradual decrease of *α* at the highest frequencies is attributed to the increasing proximity to the measurement noise floor. The extracted *β* remains close to ~1, although clear deviations occur at higher current, indicating a departure from ideal 1/*f* noise.

The observed crossover between the two current regimes may indicate a change in the dominant noise mechanism with increasing bias. In particular, the low-current regime may contain a significant contribution from carrier-number fluctuations associated with trapping and detrapping, whereas mobility fluctuations associated with impurity scattering may become increasingly important at higher currents. In the empirical Hooge model [14], the number of mobile carriers is often assumed to scale approximately linearly with current. Such an assumption is not necessarily valid for the strongly nonlinear transport in the RNPU, where the carrier population can depend nonlinearly on bias once space-charge effects due to dopants and injected carriers occur. This may provide a possible explanation for the stronger-than-quadratic current dependence observed in the high-current regime.

**Figure S9.** (a) Current noise power spectral density (PSD) $S_{I}$ for increasing bias voltage (steps of 0.2 V) in an RNPU. The current is measured between two opposing electrodes, with the remaining ones left floating. Inset: *I–V* characteristic reconstructed from the mean current $I\left( t \right)$ used for each individual PSD trace. (b) PSD normalized to $I^{2}$.

**Figure S10.** Current noise PSD $S_{I}$ as a function of mean current $I$ at selected frequencies $f$. Fits to two current regimes reveal distinct current-scaling behavior of the noise PSD.

**Figure S11.** Extracted (a) *α* as a function of frequency and (b) *β* as a function of current, based on Equation (4). The fitting range used to extract *α* is indicated in Figure S10.

**Equilibrium behavior of arsenic RNPUs**

In contrast to the boron-implanted devices, the electron-carrier devices implanted with arsenic show a clear relationship between the etch depth and the extracted activation energy (Figure S12a). By reducing the dopant-induced free carrier concentration via etching, the Fermi level becomes increasingly pinned close to mid-gap. The available free carriers are trapped by the interface states, leaving a lower carrier concentration that may contribute to band transport. As the sheet ionized donor density $N_{D,sheet}^{+}$ is reduced, the barrier to transport increases in height as a result of the stronger band bending (Figure S12b), and correspondingly the activation energy increases.

**Figure S12.** (a) Experimental Arrhenius plots of low-bias resistance $R \left( \Omega\right)$ for arsenic-implanted devices (*I*–*V* characteristics measured between adjacent contacts) with different etch times and corresponding apparent etch depths (20, 30, and 70 nm). The reported etch depths do not include the fluorocarbon layer (Section 4.1). (b) Equilibrium band diagrams, as in Figure 2b, for the three devices with different etch depths. The distance corresponds to the distance between the contacts, as in Figure 2b.

**Assessment of alternative charge transport mechanisms**

Other bulk charge transport mechanisms have been considered in our analysis. Contact-limited mechanisms are excluded due to the Ohmic contacts; the Ohmic *I–V* characteristics of the devices prior to dry etching also support this line of reasoning.

*The quasi-exponential regime*

Plotting the *I*–*V* characteristics on a semi-logarithmic scale is a common approach when characterizing diodes as it highlights both the rectifying nature of the device, as well as the exponential nature of the diffusion current. The TLM structures show that the *I*–*V* characteristic between two terminals is non-rectifying (Figure S13), which is to be expected for a *p*⁺*pp*⁺ structure, assuming that there are no other contacts that break this symmetry. Considering the energy barrier that has formed across the device is thermally activated with $|e|\phi_{B}\sim E_{a}$ and that the charge density, and consequently, the current is related exponentially to the applied voltage:

$$\begin{aligned} I\propto\exp\left( -\frac{E_{a}}{k_{B}T}+\frac{\left| e \right|V}{\eta k_{B}T} \right). \#(S1) \end{aligned}$$

Extracting the slope in the exponential-like regime ($T = 300 K$) yields ideally factors $\eta>2$, which indicates that a pure diffusion or recombination current, or a combination of the two currents, does not adequately describe transport in this regime [8].

**Figure S13.** Semi-logarithmic plot of *I*–*V* characteristics in a boron-implanted TLM structure (etch depth = 70 nm) for various $L$.

We also investigate the physical origin of this regime using TCAD simulations (Figure S14). The hole concentration $p$, assuming that the Fermi level is several $k_{B}T$ away from the valence band edge and Boltzmann statistics can be used, is given by Equation (9). Equating this to the net doping $\left| N_{A}^{-}-N_{D}^{+} \right|$ yields the difference of the Fermi level and the valence band edge $E_{\mathrm{Fp}}-E_{v}$ at which the space-charge effect occurs, and thus the onset of SCLCs is expected, given that the drift current contribution dominates the overall current density:

$$\begin{aligned} E_{\mathrm{Fp}}-E_{v}\left( p=N_{A}^{-}-N_{D}^{+} \right)=k_{B}T \ln\left( \frac{N_{v}}{\left| N_{A}^{-}-N_{D}^{+} \right|} \right). \#(S2) \end{aligned}$$

At 300 K, given $N_{v}=1.021\times{10}^{19} cm^{-3} ADDIN ZOTERO\_ITEM CSL\_CITATION \{"citationID":"ofFNMvi5","properties":\{"unsorted":false,"formattedCitation":" [10]","plainCitation":" [10]","noteIndex":0\},"citationItems":[\{"id":446,"uris":["http://zotero.org/users/local/xnNBT3wC/items/5JCWCIPP","http://zotero.org/users/20590522/items/5JCWCIPP","http://zotero.org/users/20590522/items/ZIEAQR3E"],"itemData":\{"id":446,"type":"software","citation-key":"VictoryDevice","publisher":"Silvaco, Inc.","publisher-place":"Santa Clara, CA, USA","title":"Victory Device (Version 1.24.0.R)","URL":"https://silvaco.com/","issued":\{"date-parts":[["2024",4]]\}\}\}],"schema":"https://github.com/citation-style-language/schema/raw/master/csl-citation.json"\}$ [10], $N_{D}^{+}={10}^{15} cm^{-3}$ and $N_{A}^{-}\approx{5.7\times10}^{14} cm^{-3}$ (both $N_{A}^{-}$ and $E_{\mathrm{Fp}}-E_{v}$ are extracted in the center of the device at a depth where the hole concentration is largest), this corresponds to $E_{\mathrm{Fp}}-E_{v}=261 \mathrm{meV}$ (see black dashed line in Figure S14). This condition should hold across the entire device to speak of the current being space-charge-limited.

While the strong modulation of the electrostatic potential and thus of $E_{\mathrm{Fp}}-E_{v}\left( V \right)$ resembles an exponential dependence in the *I*–*V* characteristics, the nonlinear nature of $E_{\mathrm{Fp}}-E_{v}$ below $p=\left| N_{A}^{-}-N_{D}^{+} \right|$ rules out exponential behavior corresponding to a pure diffusion current. Figure S14 additionally highlights that the voltage at which the transition to the SCLC regime occurs (based on the condition $p=\left| N_{A}^{-}-N_{D}^{+} \right|$) is indeed well approximated by the crossover voltage $V_{\mathrm{cross}}=V\left. \right|_{\max\left( m \right)}$ (here equal to ~1.1 V). The depth at which the carrier concentration is maximum thus provides an estimate of the region where most of the current flows; however, small deviations (also as a function of channel length) introduce a systematic error in the extraction of $N_{\mathrm{eff}}$ in Figures 3c, 4a, S20b, and S21.

**Figure S14.** TCAD simulation of $E_{\mathrm{Fp}}-E_{v}$ (left axis) and the power-law exponent (right axis) versus voltage *V* on linear scale for a *p*-type TLM structure ($L=300 \mathrm{nm}, N_{D}^{+}={10}^{15} cm^{-3}$). The value of $E_{\mathrm{Fp}}-E_{v}\left( V \right)$ at which the free hole concentration $p$ is equal to $\left| N_{A}^{-}-N_{D}^{+} \right|$ is represented by the black dotted line. $E_{\mathrm{Fp}}-E_{v}\left( V \right)$ is taken in the center of the device, 150 nm below the etched surface. The crossover voltage $V_{\mathrm{cross}}=1.1 V$. The light-green dashed line shows that $E_{\mathrm{Fp}}-E_{v}$ depends nonlinearly on $V.$

*Variable-range hopping*

The nonlinear nature of the current-voltage characteristics in the treated samples was originally fully attributed to a two-dimensional VRH mechanism close to the silicon surface due to an extracted $T^{-\frac{1}{3}}$ dependence of the resistance [9]. While this was explained by hopping along dopants, given the nature of the silicon surface, hopping along interface states should also not be excluded. However, further measurements and analysis on treated samples could not conclusively establish any particular temperature dependence that would point toward nearest-neighbor hopping or VRH (Figure S15). Fitting in an identical window as in Ref. [9] shows strong correlations for all temperature dependencies; similar analysis and conclusions can be made with the available data from Ref. [9]. Thus, we can speak of Arrhenius behavior, as well as an apparent transition to a freeze-out regime with decreasing temperature, but hopping mechanisms cannot be proven with certainty.

**Figure S15.** Semi-logarithmic plot of low-bias resistance $R (\Omega)$ as a function of (a) $T^{-1}$, (b) $T^{-\frac{1}{2}}$, (c) $T^{-\frac{1}{3}}$, and (d) $T^{-\frac{1}{4}}$. The coefficient of determination is given in each plot, with $SS_{\mathrm{res}}$ and $SS_{\mathrm{tot}}$ the residual and total sum of squares, respectively. The sample treatment involved an HF wet etch for 2 minutes, followed by an anneal at $250 ˚C$ for $5$ minutes.

*Poole-Frenkel emission*

An alternative mechanism such as Poole–Frenkel emission [8, 15] across the interface traps was considered:

$$\begin{aligned} J_{s}\propto\mathcal{E}\exp\left[ \frac{-\left| e \right|\left( \phi_{B,PF}-\sqrt{\frac{\left| e \right|\mathcal{E}}{\pi\epsilon}} \right)}{k_{B}T} \right], \#(S3) \end{aligned}$$

where $J_{s}$ is the surface current density flowing along the interface traps and $\phi_{B,PF}$ is the barrier height which corresponds to the depth of the traps’ potential well. The *I*–*V* characteristics of the TLM structure in Figure 3 are re-illustrated in the form of Poole–Frenkel plots (Figure S16a), assuming a uniform electric field $\mathcal{E}=V/L$ across the device. Fits performed for both intermediate fields and in the high-field limit resulted in either physically non-viable dielectric constants ($\epsilon_{r}$ < 1), or permittivities exceeding that of silicon (Figure S16b). Furthermore, given the dimensions of both the devices and test structures, Poole–Frenkel emission is unlikely to be the dominant transport mechanism. This model also assumes the absence of space-charge effects and therefore a uniform electric field, whereas the present analysis indicates that space-charge effects are present in the device.

The strong experimental evidence, supported by TCAD simulations, indicating a bulk conduction mechanism reduces the likelihood of charge transport being dominated by mechanisms based on localized surface states, such as Poole–Frenkel emission or 2D VRH.

**Figure S16.** (a) Poole–Frenkel plots of untreated boron-implanted TLM structure, plotted for various $L$. The current density is defined as a surface current density $J_{s}$, normalized to $\text{26 }\text{μm}$, i.e., the width of the TLM structure (Figure S1). The average electric field $\mathcal{E=}V /L$ is assumed to be uniform across the device. (b) Poole–Frenkel plot for a 400 nm channel, including fits in an intermediate-field regime ($\epsilon_{r}=0.4$) and in the high-field limit ($\epsilon_{r}=19$).

**Influence of mobility on simulated *I*–*V* characteristics**

A deeper understanding of the length dependence of the current density can be obtained from TCAD simulations. Normalizing the *I*–*V* characteristics to different channel lengths (Figure S17) supports the experimental finding (Figure 3b) that, in the SCLC regime, the current density scales as $L^{-1}$. This behavior justifies the inclusion of the Caughey–Thomas mobility model to account for field-dependent mobility.

In contrast, assuming a field-independent mobility leads to a current density scaling of $L^{-2}$ in the SCLC regime (Figure S18), as predicted by Equation (10). In this case, the quadratic voltage dependence of the Mott–Gurney law is recovered (Figure S19).

**Figure S17.** (a) *I–V* characteristics of simulated *p*-type TLM structure including all relevant mobility models, normalized to (b) $L$, (c) $L^{2}$, and (d) $L^{3}$. The width of the device, which scales the current, is assumed to be 20 nm large. In the high-field regime, the current scales with $L^{-1}$, supporting the theory on SCLC modified by velocity saturation, given by Equation (12), and the experimental results.

**Figure S18.** (a) *I–V* characteristics of simulated *p*-type TLM structure excluding the Caughey–Thomas mobility model, normalized to (b) $L$, (c) $L^{2}$, and (d) $L^{3}$. The width of the device is assumed to be 20 nm large. Since the mobility is independent of the electric field, in the high-field SCLC regime, the current scales with $L^{-2}$, in line with Equation (10).

**Figure S19.** Simulated power-law exponent versus voltage in a *p*-type TLM structure ($L=300 \mathrm{nm}$). The inclusion of mobility models, in particular the Caughey–Thomas mobility model, modifies the voltage dependence such that in the high-field limit, $I\propto V$ instead of $\propto V^{2}$, supporting Equations (10–12).

**Figure S20.** (a) TLM *I–V* characteristics on an arsenic-implanted sample with an apparent etch depth equal to 50 nm (etch time = 3 min, $L_{c}=500 \mathrm{nm}$). (b) Plot of crossover voltages $V_{\mathrm{cross}}$ versus $L^{2}$ for two test structures with the geometry described in (a). Based on Equation (14), $N_{\mathrm{eff}}=3.5\times{10}^{15} cm^{-3}$ is extracted. Inset: Local power-law exponent $m\left( V \right)$ for varying channel lengths. An outlier for one 1200 nm channel is excluded from the fit; substrate charging effects may have heavily skewed the crossover voltage prior to and during the measurement.

**Influence of various parameters on space-charge effect**

*Geometry*

We investigate how the contact length $L_{c}$, i.e., the length of the metal in the *x*-direction (along the channel), as in Figure 4b, influences the crossover voltage, and thus the electrostatics in the device. Figure S21 shows a clear trend of the crossover voltage decreasing with increasing $L_{c}$. Due to the higher concentration of carriers injected into the device for the longer contacts, the condition for which $p>\left| N_{A}^{-}-N_{D}^{+} \right|$ shifts to lower voltages. This therefore also influences the extraction of $N_{\mathrm{eff}}$, which decreases with increasing $L_{c}$. We thus conclude that the geometry of the contacts is central to how space charge is spread across the device. This also expresses the need for a three-dimensional model in order to adequately describe the electrostatics in RNPUs.

**Figure S21.** Simulated plots of $V_{\mathrm{cross}}$ versus $L^{2}$ ($N_{D}^{+}={10}^{15} cm^{-3},N_{\mathrm{tp}}={10}^{12} cm^{-2} eV^{-1}$) for various contact lengths $L_{c}$ in a *p*-type TLM structure. The extracted $N_{\mathrm{eff}}$ values are indicated alongside the corresponding fitted lines. Inset: Local power-law exponent $m$ versus $V$ for three contact lengths ($L=600 \mathrm{nm}$).

*Background doping*

TCAD simulations also allow us to observe how the background doping influences the device nonlinearity. Figure S22a shows that the crossover voltage increases with increasing doping concentration, as does the degree of nonlinearity. Since the net doping increases, the depletion region formed at the *p*-*n* junction shifts upwards, decreasing the equilibrium hole concentration. The injected holes must counteract more dopants across the device such that the voltage at which the SCLC regime is entered naturally shifts to higher voltages. The sub-Ohmic behavior observed for $N_{D}^{+}={10}^{16} cm^{-3}$ can be explained by impurity and carrier-carrier scattering effects, accounted for by the low-field Klaassen mobility model [10, 16]. When the background doping is *p*-type rather than *n*-type, the nonlinearity is significantly suppressed. Due to the increased equilibrium hole concentration and no depletion region at the *p*-*n* junction to set a low hole concentration, the transition to the velocity-saturation SCLC regime is gradual without any significant rise in current. This is experimentally validated in Figure S22b by fabricating TLM structures identical to those analyzed in Figure 3, but on a *p*-type substrate ($N_{A}^{-}$ $\sim$ $2\times{10}^{15}\text{ }cm^{-3}$). The resulting devices exhibit significantly higher currents compared to those in Figure 3 and show a complete absence of the quasi-exponential transport regime.

**Figure S22.** (a) Simulated local power-law exponent versus voltage for various background doping concentrations in a *p*-type TLM structure ($L=300 nm, L_{c}=200 nm,N_{\mathrm{tp}}={10}^{12} cm^{-2} eV^{-1}$). Replacing the *n*-type background doping with *p*-type doping strongly suppresses the nonlinear response. The dashed box highlights the suppressed nonlinearity for a *p*-type background doping concentration of ${10}^{15} cm^{-3}$. (b) Experimental validation of the simulated behavior in (a). Log–log plot of *I*–*V* characteristics for a TLM structure ($L_{c}=200 \mathrm{nm}$) fabricated identically to the devices in Figure 3, but using a *p*-type substrate. Inset: Local power-law exponent $m$ versus $V$ for various *L*.

*Implanted doping profile*

In all TCAD simulations we utilize the dopant profile based on SIMS measurements, and assume that an effective fraction of dopants to be electrically active. We assume in TCAD simulations that after RTA, only 20% of dopants are electrically active, and that after RIE, only 5% of the dopants in the channel region are electrically active. Though it is unlikely that the percentage of active dopants is constant versus depth, the analysis here still provides qualitative insight into the necessary assumption of dopant deactivation. Furthermore, based on our analysis in Section 4.1, it is more likely that closer to 100% of dopants are active below the contacts, and that less than 1% of dopants are active in the RIE-exposed channel region. The assumed profile in TCAD thus acts as an effective active-dopant profile, constrained by the measured crossover voltages. We thus analyze the sensitivity of the space-charge effect [8] on the implanted dopant profile, in other words, on $N_{A}^{-}$, to evaluate the role of dopant deactivation. Note that in the TCAD simulations we assume that ${N_{A}=N}_{A}^{-}$, which does not affect the analysis on the space-charge effect, but only influences the modeling of carrier mobility, which we do not focus on here.

Figure S23a shows that a finite degree of dopant deactivation must be assumed in order to reproduce the experimental observations. The ionized acceptor concentration in the plasma-exposed channel region is crucial (Figure S23b,c), and both $m\left( V \right)$ and $V_{\mathrm{cross}}$ are sensitive to the availability of holes, particularly in the channel. In principal, $N_{A}^{-}$ should be ~$N_{D}^{+}$ in the bulk region where the hole concentration is highest, i.e., away from the trap-dense surface where carriers are immobilized. Fundamentally, $p_{\mathrm{eq}}$ must be sufficiently low for the space-charge effect to occur; both surface states and dopant deactivation, which are linked to the RIE process, play an important role in this regard.

**Figure S23.** TCAD simulations investigating deactivation of implanted dopants. Simulations are performed for a *p*-type TLM structure ($L=300 \mathrm{nm}, L_{c}=200 nm, N_{D}^{+}={10}^{15} cm^{-3}, N_{\mathrm{tp}}={10}^{12} cm^{-2} eV^{-1}$), assuming different combinations of dopant deactivation under the contacts / in the channel region. Local power-law exponent is plotted (a) assuming the implanted profile is spatially uniform across the device, (b) accounting for varying degrees of dopant deactivation beneath the contacts, and (c) accounting for varying degrees of dopant deactivation in the channel region. (d) The equilibrium hole concentration $p_{\mathrm{eq}}$ (solid line), the ionized (implanted) acceptor concentration $N_{A}^{-}$ (dashed line) and the ionized (background) donor concentration $N_{D}^{+}$ (dotted line) are plotted assuming all dopants are active beneath the contacts, and for varying fractions of active dopants in the plasma-exposed channel region. The concentrations are taken in the center of the device, 130 nm below the etched surface, similarly to the approach taken in Figure 4b.

*Substrate potential*

TCAD simulations support the observation in Ref. [9] that nonlinearity can be introduced to devices with $m \sim1$ by applying a positive substrate potential on the *n*-type substrate of boron RNPUs (Figure S24a). TCAD simulations show that the positive substrate potential reduces the free hole concentration in the bulk, increasing the depletion width at the *p*-*n* junction between the implanted region and the *n*-doped substrate (Figure S24b). Due to the lower potentials on the input and output electrodes, holes move toward the contacts, decreasing the equilibrium hole concentration $p_{\mathrm{eq}}$ in the bulk (Figure S24c). The reduction in $p_{\mathrm{eq}}$ accounts for the observed shift of $V_{\mathrm{cross}}$ to higher voltages with increasing $V_{\mathrm{sub}}$, as shown in Figure S24a. Notably, $\frac{\partial p}{\partial\mathcal{l}}$ increases with increasing $V_{\mathrm{sub}}$, which signifies a stronger contribution of the diffusion current to the current density. This supports our discussions and analysis in Sections 2.3 and 2.4 that point towards a large role of diffusion current in the quasi-exponential regime. The substrate potential can thus be seen as effectively enhancing the internal electric field counteracting carrier injection, leading to a strongly nonuniform electric field profile which facilitates steep carrier concentration gradients, manifesting as a pronounced nonlinear dependence of current density on voltage.

**Figure S24.** TCAD simulations investigating the influence of the substrate potential on the *I–V* characteristics of RNPUs. (a) *I–V* characteristics for a floating substrate (3 μm thick, not shown here) and increasing substrate voltages $V_{\mathrm{sub}}$ ($L=300 \mathrm{nm}, L_{c}=200 nm, N_{D}^{+}={10}^{15} cm^{-3}, N_{\mathrm{tp}}={10}^{12} cm^{-2} eV^{-1}$). Inset: Local power-law exponent $m\left( V \right)$ for varying $V_{\mathrm{sub}}$. (b) Free hole concentration *p* with voltage $V_{\mathrm{in}}=1 V$ on the sourcing input electrode, $V_{\mathrm{out}}=0 V$ on the grounded output electrode, and $V_{\mathrm{sub}}=10 V$ on the substrate. Hole current flows along path $\mathcal{l}$. (c) Hole concentration $p$ along path $\mathcal{l}$ in (b) for increasing $V_{\mathrm{sub}}$. Solid and dashed lines indicate $p$ at $V_{\mathrm{in}}=0 V$ and $V_{\mathrm{in}}=1 V$, respectively.

### **References**

1. C. Lee, C. Lee, S. Lee, J. Choi, H. Yoo, and S. G. Im. A Reconfigurable Binary/Ternary Logic Conversion-In-Memory Based On Drain-Aligned Floating-Gate Heterojunction Transistors. *Nature Communications*, *14*, no. 1, 3757, 2023. https://doi.org/10.1038/s41467-023-39394-5.

2. R. Berdan, T. Marukame, K. Ota, et al. Low-Power Linear Computation Using Nonlinear Ferroelectric Tunnel Junction Memristors. *Nature Electronics*, *3*, no. 5, 259–266, 2020. https://doi.org/10.1038/s41928-020-0405-0.

3. T. Günkel, E. Miranda, L. Balcells, N. Mestres, A. Palau, and J. Suñé. Trap-Controlled Conduction And Metal–Insulator Transition In Superconducting Cuprate Memristors. *ACS Applied Electronic Materials*, *8*, no. 3, 1099–1107, 2026. https://doi.org/10.1021/acsaelm.5c02017.

4. P. Zhu, C. Zhang, J. Yang, et al. Dielectric Stacking-Engineered Scalable Reconfigurable Transistor Platform For Adaptive Logic Circuits. *Nature Communications*, *17*, no. 1, 9028, 2026. https://doi.org/10.1038/s41467-026-75864-2.

5. R. Peng, Y. Wu, B. Wang, et al. Programmable Graded Doping For Reconfigurable Molybdenum Ditelluride Devices. *Nature Electronics*, *6*, no. 11, 852–861, 2023. https://doi.org/10.1038/s41928-023-01056-1.

6. M.-P. Lee, C. Gao, M.-Y. Tsai, et al. Silicon–Van Der Waals Heterointegration For CMOS-Compatible Logic-In-Memory Design. *Science Advances*, *9*, no. 49, eadk1597, 2023. https://doi.org/10.1126/sciadv.adk1597.

7. P. J. Eaton, and P. West. Atomic Force Microscopy. 2010.

8. S. M. Sze, and K. K. Ng. Physics Of Semiconductor Devices. 2007.

9. T. Chen, J. van Gelder, B. van de Ven, et al. Classification With A Disordered Dopant-Atom Network In Silicon. *Nature*, *577*, no. 7790, 341–345, 2020. https://doi.org/10.1038/s41586-019-1901-0.

10. Victory Device (Version 1.24.0.R). Silvaco, Inc. Santa Clara, CA, USA. 2024. Available at: https://silvaco.com/.

11. C. R. Helms, and E. H. Poindexter. The Silicon-Silicon Dioxide System: Its Microstructure And Imperfections. *Reports on Progress in Physics*, *57*, no. 8, 791–852, 1994. https://doi.org/10.1088/0034-4885/57/8/002.

12. P. M. Lenahan, T. D. Mishima, J. Jumper, T. N. Fogarty, and R. T. Wilkins. Direct Experimental Evidence For Atomic Scale Structural Changes Involved In The Interface-Trap Transformation Process. *IEEE Transactions on Nuclear Science*, *48*, no. 6, 2131–2135, 2001. https://doi.org/10.1109/23.983184.

13. P. Welch. The Use Of Fast Fourier Transform For The Estimation Of Power Spectra: A Method Based On Time Averaging Over Short, Modified Periodograms. *IEEE Transactions on Audio and Electroacoustics*, *15*, no. 2, 70–73, 1967. https://doi.org/10.1109/TAU.1967.1161901.

14. F. N. Hooge. 1/F Noise Sources. *IEEE Transactions on Electron Devices*, *41*, no. 11, 1926–1935, 1994. https://doi.org/10.1109/16.333808.

15. R. M. Hill. Poole-Frenkel Conduction In Amorphous Solids. *Philosophical Magazine*, *23*, no. 181, 59–86, 1971. https://doi.org/10.1080/14786437108216365.

16. D. B. M. Klaassen. A Unified Mobility Model For Device Simulation—I. Model Equations And Concentration Dependence. *Solid-State Electronics*, *35*, no. 7, 953–959, 1992. https://doi.org/10.1016/0038-1101(92)90325-7.
